# Nhp2 is a reader of H2AQ105me and part of a network integrating metabolism with rRNA synthesis

**DOI:** 10.15252/embr.202152435

**Published:** 2021-08-19

**Authors:** Julia S P Mawer, Jennifer Massen, Christina Reichert, Niklas Grabenhorst, Constantine Mylonas, Peter Tessarz

**Affiliations:** ^1^ Max Planck Research Group “Chromatin and Ageing” Max Planck Institute for Biology of Ageing Cologne Germany; ^2^ Cologne Excellence Cluster on Stress Responses in ageing‐associated Diseases (CECAD) Cologne Germany; ^3^ Present address: Novartis Institutes for BioMedical Research Cambridge MA USA

**Keywords:** chromatin, epigenetic reader, ribosome biogenesis, rRNA, small subunit processome, Chromatin, Transcription & Genomics, RNA Biology, Signal Transduction

## Abstract

Ribosome biogenesis is an essential cellular process that requires integration of extracellular cues, such as metabolic state, with intracellular signalling, transcriptional regulation and chromatin accessibility at the ribosomal DNA. Here, we demonstrate that the recently identified histone modification, methylation of H2AQ105 (H2AQ105me), is an integral part of a dynamic chromatin network at the rDNA locus. Its deposition depends on a functional mTor signalling pathway and acetylation of histone H3 at position K56, thus integrating metabolic and proliferative signals. Furthermore, we identify a first epigenetic reader of this modification, the ribonucleoprotein Nhp2, which specifically recognizes H2AQ105me. Based on functional and proteomic data, we suggest that Nhp2 functions as an adapter to bridge rDNA chromatin with components of the small subunit processome to efficiently coordinate transcription of rRNA with its post‐transcriptional processing. We support this by showing that an H2AQ105A mutant has a mild defect in early processing of rRNA.

## Graphical abstract

The rDNA‐specific histone modification H2AQ105me depends on H3K56 acetylation and is read by Nhp2. Together they play a role in mediating recruitment of the small subunit processome to rDNA.

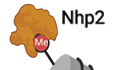

## Introduction

Regulation of ribosomal biogenesis is a complex process. It requires all three eukaryotic polymerases and is tightly linked to the cell cycle (Bernstein *et al*, 2007; Piazzi *et al*, 2019) and metabolic state, particularly via Tor‐mediated signalling (Mayer & Grummt, 2006; Huber *et al*, 2011). It was noted early on that inhibition of Tor kinase by rapamycin leads to shut‐down of ribosomal RNA (rRNA) transcription by RNA polymerase I (RNA Pol I) and concomitant decrease in the levels of acetylation of lysine 5 and 12 on histone H4, suggesting that chromatin is not only involved in the regulation of RNA Pol I, but that chromatin profile of the rDNA can also be used as a read‐out for RNA Pol I activity (Tsang *et al*, 2003). H3K56 acetylation is another modification that has been described to be dependent on Tor signalling (Chen *et al*, 2012). In particular, histone acetylation seems to be inhibited by rapamycin—presumably as it is a mark for actively transcribed chromatin.

Transcription of rRNA is followed by several post‐transcriptional processing steps including base and ribose methylation or pseudouridylation of the rRNA. Base methylation occurs late in ribosome maturation and only in highly conserved rRNA sequences. The majority of ribose methylation takes place early in rRNA processing and is considered to be important for rRNA folding or association with chaperone proteins that might help folding of rRNA. Single sites of ribose methylation are not essential (Weinstein & Steitz, 1999), but global rRNA demethylation severely impairs growth (Tollervey *et al*, 1993). Pseudouridylation is thought to play a variety of roles in the ribosome, including the improvement of translational fidelity (Jack *et al*, 2011).

Pioneering studies using electron microscopy demonstrated that rRNA folds co‐transcriptionally (Scull & Schneider, 2019). Importantly, RNA Pol I transcription speed and folding kinetics are directly coupled as shown by mutations in RNA Pol I that decrease the rate of synthesis (Schneider *et al*, 2007). Not only does lower transcription speed impact rRNA folding, but it also directly affects rRNA processing (Duss *et al*, 2018). In addition, previous work showed that early processing steps, particularly of the small ribosomal subunit components also occur co‐transcriptionally (Gallagher *et al*, 2004; Kos & Tollervey, 2010). Taken together, these data suggest a tight link between chromatin architecture and transcriptional regulation of the rDNA and synthesis and processing of the rRNA. One potential way of linking these different steps in an efficient way would be to directly recruit the rRNA processing machinery to the rDNA chromatin. We previously identified the methylation of H2AQ105 as an RNA Pol I dedicated histone modification that is exclusively enriched at the rDNA locus (Tessarz *et al*, 2014). The modification is dependent on RNA Pol I‐mediated transcription and is catalysed by the nucleolar methyltransferase NopI/Fibrillarin (Tessarz *et al*, 2014; Loza‐Muller *et al*, 2015; Iyer‐Bierhoff *et al*, 2018).

Here we show that H2AQ105me is an integral part of a dynamic chromatin network at the rDNA locus. Deposition of the modification in yeast is cell cycle dependent—similar to what was observed in human cells (Iyer‐Bierhoff *et al*, 2018)—and relies on proliferation. Furthermore, we can demonstrate that the methylation is downstream of the Tor signalling pathway and acetylation of histone H3 at position K56. We identify Nhp2 as an epigenetic reader of H2AQ105me and provide evidence that Nhp2 might function as an adapter to bridge the rDNA chromatin with components of the ribosome’s small‐subunit (SSU) processome.

## Results and Discussion

### H2AQ105 methylation is a dynamic histone modification that requires cell proliferation and depends on Tor signalling and H3K56 acetylation

**Figure 1:**
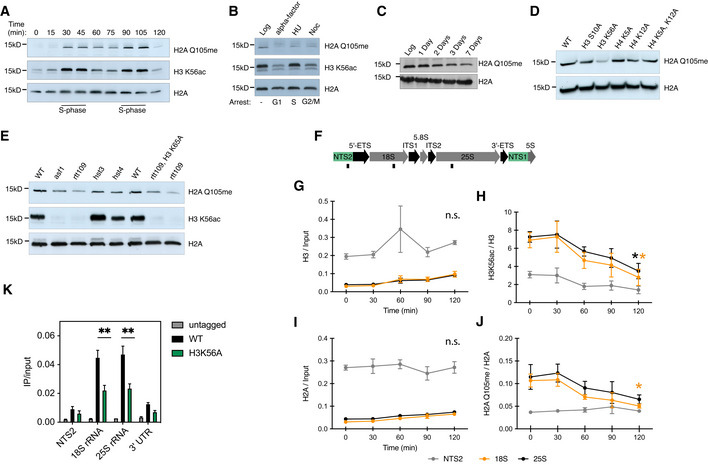
H2AQ105me fluctuates with the cell cycle and is a mark of proliferating cells ACell cycle profile of H2AQ105me. Logarithmically growing cells were arrested using alpha factor for 2.5 h. Upon release (*t* = 0), samples were taken every 15 min and samples were analysed for H2AQ105me levels using Western blotting. S‐phase is indicated by the enrichment of H3K56ac.BH2AQ105me levels upon arrest (2.5 h) at different cell cycle stages. Treatment is given above the lanes.CH2AQ105me levels upon entry of cells into stationary phase.DH2AQ105me levels in various histone mutations targeting sites of well‐known cell cycle‐dependent modifications.EH2AQ105me and H3K56ac levels in deletions of genes important for H3K56ac deposition (see text for details).FScheme of rDNA locus and indication of primer locations used in G–J.G–JChIP‐qPCR for indicated antibodies after treatment with 5 nM rapamycin for the indicated time points. Samples were either normalized to input or to the core histone. *n* = 3, biological replicates, error bars are standard error of the mean. *P < 0.05, relative to *t* = 0, based on unpaired, 2‐tailed *t*‐test.KChIP‐qPCR for Nop1‐FLAG using anti‐FLAG tag antibody. Samples were normalized using input. *n* = 3, biological replicates, and error bars are standard error of the mean. **P < 0.001, based on unpaired, 2‐tailed *t*‐test. Source data are available online for this figure.

As ribosome biogenesis is tightly linked to cellular state, we initially sought to determine whether H2AQ105me levels, in the yeast *Saccharomyces cerevisiae*, were regulated in a cell cycle dependent manner. Cells were arrested in G1 using alpha factor. Following release of G1 arrest, cells were sampled at time intervals and levels of H2AQ105me were assessed by western blotting (Fig 1A). H2AQ105me levels fluctuate throughout the cell cycle and peak in S‐phase, as shown by the co‐increase of H2AQ105me and the S‐phase marker, H3K56ac. These data confirm observations in human cells, in which a cell cycle‐dependent fluctuation of H2AQ105me was reported (Iyer‐Bierhoff *et al*, 2018). Interestingly, arresting the cell cycle in any phase leads to loss of H2AQ105me, indicating that proliferation itself is required for the methylation (Fig 1B). It is important to note that prolonged G1 arrest leads to a continuous opening of the rDNA locus, while it leads to lower recruitment of RNA Pol I (Wittner *et al*, 2011). In addition, there is very little rRNA synthesis in G2/M as chromatin compacts before cell division, which is visible in nascent RNA labelling experiments (Iyer‐Bierhoff *et al*, 2018). Even an arrest in S‐phase using the drug hydroxyurea (HU), which depletes the cell of deoxynucleotides leading to DNA replication fork stalling and collapse, leads to loss of H2AQ105 methylation rather than its accumulation, as is seen for H3K56ac (Fig 1B). This observation is likely explained by a recent study in which rDNA transcription dynamics were studied under HU‐induced cell cycle arrest, showing that rDNA transcription, as well as DNA replication, is halted by HU (Charton *et al*, 2019). This strengthens the observation that RNA Pol I‐mediated transcription of the rDNA is required for deposition of H2AQ105me and that the methylation is associated with transcribed rDNA repeats. To explore the requirement for proliferation further, yeast cells were grown deep into stationary phase (Fig 1C) by growing in a sealed flask for up to one week without replenishing the growth medium or diluting the density of cells. Cells were sampled initially during log‐phase as a reference point and then at days 1, 2, 3 and 7. Western blot analysis shows a progressive decrease in levels of H2AQ105me over time.

The observation that H2AQ105me levels cycled with cell cycle progression prompted us to address if other histone residues known to be modified in a cell cycle dependent manner would crosstalk with H2AQ105me. Interestingly, mutating H3K56 to alanine led to a strong reduction of H2AQ105me, while other mutations tested did not have an effect on the level of H2AQ105me (Fig 1D). To corroborate the dependence of H2AQ105me on H3K56ac, we went on to analysed the requirement for the H3K56 acetylation machinery. Before deposition into chromatin, H3 is bound by the histone chaperone Asf1 (Chen *et al*, 2008). The histone acetyl‐transferase Rtt109 subsequently binds the Asf1‐H3/4 complex and acetylates H3 at K56 (Chen *et al*, 2008) prior to the incorporation of the H3/4 tetramer into chromatin. Deletions of both *asf1* and *rtt109*, also led to a reduction of H2AQ105me confirming the result of the mutational analysis in H3 (Fig 1E). Rtt109 is a slow growing strain, and this reduction in growth might be responsible for the reduction in H2AQ105me. However, growth is rescued by the additional mutation in H3K56A (Han *et al*, 2007). Despite this growth rescue, H2AQ105me levels remain low in this double mutant (Fig 1E), indicating that acetylation at H3K56 rather than growth rate itself is important for H2AQ105 methylation. H3K56ac is de‐acetylated by Hst3/4 (Kaplan *et al*, 2008). In line with the idea that acetylation of H3K56 is required for H2AQ105me, deletions of *hst3/4* did not change the level of H2AQ105me (Fig 1E).

**Figure EV1:**
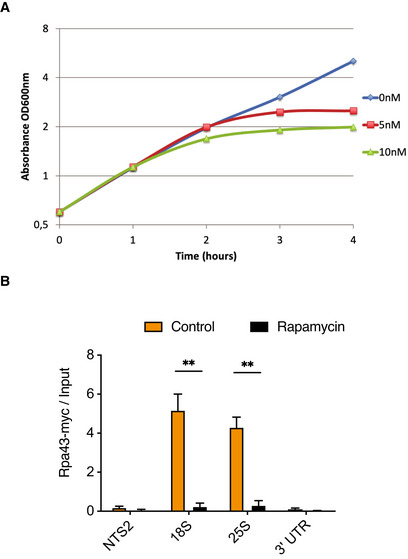
Rapamycin reduces RNA Pol I transcription of rDNA Growth curve of WT yeast cells challenged with indicated concentrations of rapamycin.Loss of RNA Pol I occupancy at the rDNA upon treatment with 5 nM rapamycin. *n* = 3, biological replicates, error bars are standard error of the mean. **P < 0.001, based on unpaired, 2‐tailed *t*‐test.

To further investigate the crosstalk between H2AQ105me and H3K56ac, we addressed the role of mTor signalling on both modifications. It is well known that mTor is a critical regulator of rDNA transcription (Mayer & Grummt, 2006). Interestingly, mTor has also been demonstrated to be involved in the regulation of H3K56 acetylation at the rDNA locus (Chen *et al*, 2012). Addition of rapamycin led to a stop in proliferation (Fig EV1A) and a loss of RNA Pol I from the rDNA locus (Fig EV1B). Using chromatin immunoprecipitation (ChIP), we confirmed the described decrease in H3K56 acetylation at the rDNA upon inhibition of mTor using Rapamycin (Chen *et al*, 2012) (Fig 1F–H). We then performed ChIPs using an H2AQ105me specific antibody (Tessarz *et al*, 2014). As with H3K56ac, we observed a time‐dependent loss of H2AQ105me, which was restricted to the coding region of the rDNA (Fig 1I and J). Interestingly, in both cases, we did not observe an increase in the deposition of the core histone (Fig 1G and I). Finally, we wanted to address how acetylation of H3K56 mediates H2AQ105me. As shown previously, substituting H3K56 for alanine as well as an *asf1* deletion led to a reduction of Pol I occupancy at the rDNA (Chen *et al*, 2012). Thus, it is plausible that the observed dependency of H2AQ105me on H3K56ac is due to a reduction in transcription rate and a concomitant decrease in Nop1 recruitment to the rDNA. As Nop1 is the methyltransferase responsible for H2AQ105 methylation (Tessarz *et al*, 2014; Loza‐Muller *et al*, 2015; Iyer‐Bierhoff *et al*, 2018), we used a FLAG‐tagged version of Nop1 and performed ChIP using a FLAG antibody to test if recruitment was reduced. Indeed, Nop1 was efficiently enriched over the transcribed region of the rDNA locus, but this enrichment was decreased in the presence of an alanine substitution at H3K56 (Fig 1K). Taken together, methylation of H2AQ105 depends on active rDNA transcription, H3K56 acetylation and the integration of metabolic cues via the mTor pathway. As H2AQ105me is exclusively localized on the rDNA, this mark might be a great cellular reporter for proliferative cells. Such a function would be similar to phosphorylation of threonine 11 in histone H3, which was recently described as an endpoint of the Sch9/Tor pathway that integrates a response to nutrient availability and ultimately, shaped chromatin to allow efficient transcription (Oh *et al*, 2018). In contrast to H2AQ105me, H3T11ph, however, is found genome‐wide, including metabolic and ribosomal protein genes, as well as rDNA (Oh *et al*, 2018).

### Ribosome biogenesis factors are enriched on H2AQ105me

**Figure 2:**
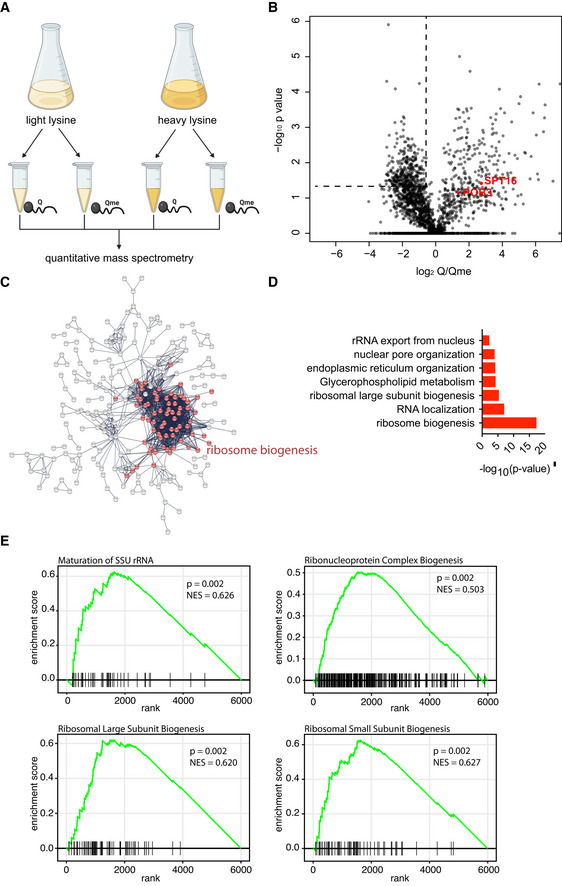
Mass spectrometry identifies ribosome biogenesis factors to be recruited to H2AQ105me Schematic of the experimental strategy.Volcano plot of quantitative SILAC experiments. Left upper quadrant indicates significantly, 2‐fold enriched proteins bound specifically to H2AQ105me. Members of the FACT complex (SPT16/POB3) are highlighted in red.STRING network of proteins enriched on H2AQ105me (highlighted in red are proteins involved in ribosome biogenesis). Protein names can be found in Dataset EV1.Gene ontology enrichment for proteins bound to H2AQ105me.Gene set enrichment analysis for the altered transcriptome in an H2AQ105A background compared to wildtype. The normalized enrichment score (NES) is reported as are individual p‐values. Vertical black lines on the x‐axis represent genes in gene sets. The green line connects points of enrichment score and genes. Enrichment score is the maximum deviation from zero as calculated for each gene going down the ranked list and represents the degree of over‐representation of a gene set at the top or the bottom of the ranked gene list.

**Figure 3:**
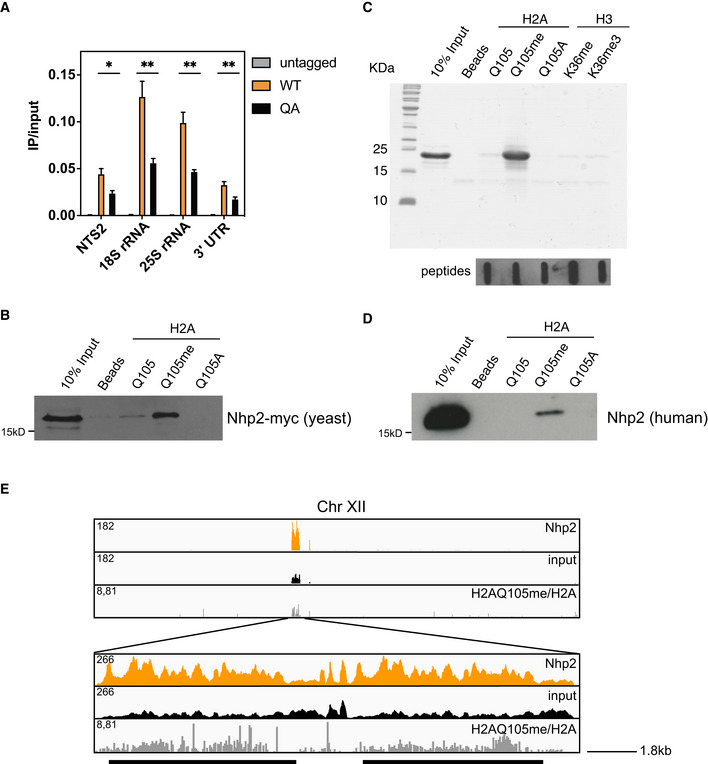
Nhp2 is an epigenetic reader of H2AQ105me ChIP‐qPCR for Nhp2‐myc using anti‐myc antibody across the rDNA locus. Samples were normalized to input. *n* = 3, biological replicates, error bars are standard error of the mean. *P < 0.05, **P < 0.001, based on unpaired, 2‐tailed *t*‐test.Peptide pulldown on H2AQ105 peptides from yeast expressing myc‐tagged Nhp2.Peptide pulldown using recombinantly purified Nhp2. Eluted peptides were slot blotted and detected using streptavidin‐HRP.Peptide pulldown on H2AQ105 peptides from lysates of human cells (HEK293T).ChIP‐seq profile of Nhp2 (orange) and its respective input (black) across the entire chromosome XII of yeast. H2A‐normalized H2AQ105me (grey) was directly taken from (Tessarz *et al*, 2014) as comparison. Magnification shows the two annotated rDNA loci in the yeast genome indicated by the black bars below the tracks. Scale bar is for the magnification. Source data are available online for this figure.

Previous work identified the region surrounding H2AQ105 as part of a recognition sequence for the histone chaperone FACT (McCullough *et al*, 2011). Methylation of Q105 prevents FACT from binding to H2A, impairing its redeposition following transcription and resulting in loss of histones at the rDNA (Tessarz *et al*, 2014). Given the link of H2AQ105me with active rRNA transcription and its demonstrated role in FACT binding inhibition, we wanted to address whether methylation of H2AQ105 could also promote binding by serving as a recognition site for the recruitment of protein complexes to the rDNA. As this histone modification is exclusively localized in the nucleolus (Tessarz *et al*, 2014; Loza‐Muller *et al*, 2015; Iyer‐Bierhoff *et al*, 2018), it would be ideally suited to serve as a chromatin beacon that marks actively transcribing RNA Pol I. To test this, we used a peptide‐pulldown approach that has been successfully performed on several histone modifications (Vermeulen *et al*, 2010). Following SILAC labelling of yeast cultures, whole cell lysis pulldowns were performed using unmodified and modified H2A peptides spanning the region of Q105 and analysis was performed by quantitative mass spectrometry (Fig 2A). As expected, we observed Pob3 and Spt16 – both subunits of the yeast FACT complex—specifically enriched on the unmodified H2A peptide (Fig 2B). 139 proteins were found to be significantly, 2‐fold or more, enriched on peptides harbouring the H2AQ105me modification (Fig 2B and Dataset EV1). Analysis of this list using the online protein–protein interaction network and functional analysis tool, String (https://string‐db.org/), showed that these proteins form a tight network (Fig 2C). Gene ontology (GO) enrichment highlighted this network to be composed of ribosome biogenesis factors and ribosomal proteins (Fig 2D). These data suggest that H2AQ105me might indeed help to recruit ribosome biogenesis factors to the site of active rDNA transcription. Because much of the rRNA processing occurs co‐transcriptionally (Turowski & Tollervey, 2015), recruitment of processing factors to the rDNA chromatin would facilitate this process. Based on these data, we hypothesized that an alanine mutant of H2AQ105 might impact binding of the identified ribosome biogenesis factors to the rDNA. As a result, an H2AQ105A mutant may be characterized by an altered gene expression programme to compensate for loss of the methylation. We performed RNA sequencing in wildtype and H2AQ105A; the changes in the transcriptome were subtle. However, we noticed changes in many snRNA transcripts, including snR11, 35, 42 and 85, which are H/ACA box small nucleolar rRNAs that guide pseudouridylation of the rRNA within ribosomal subunits (Schattner *et al*, 2004). To corroborate this finding, we analysed the data using gene set enrichment (GSEAMootha *et al*, 2003; Subramanian *et al*, 2005)) and identified several enriched categories, including some for ribosome and SSU processome biogenesis (Fig 3E), indicating that an H2AQ105A strain does exhibit some compensatory mechanisms. The fact that snRNAs are amongst the de‐regulated transcripts, and thus, ribosome biogenesis pathways are enriched in the GSEA support the role of H2AQ105me in ribosomal RNA transcription and potentially processing as well.

### Nhp2 is a reader of H2AQ105me

**Figure EV2:**
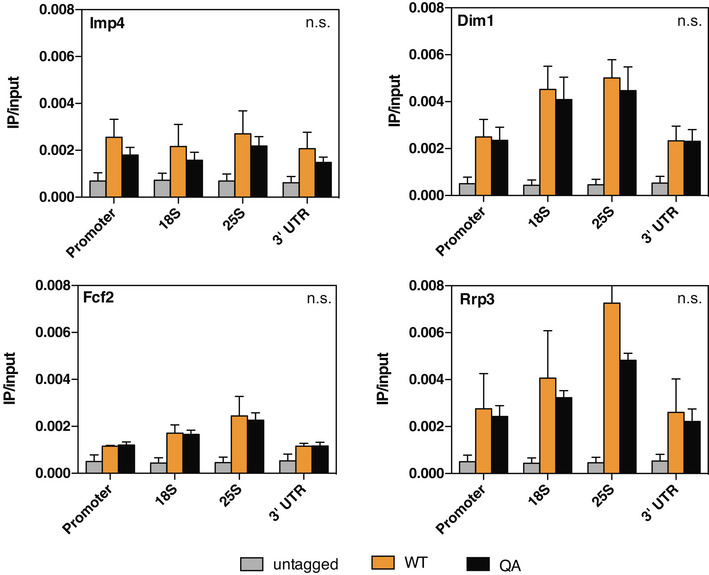
ChIP‐qPCR analysis of potential H2AQ105me readers ChIP‐qPCR analysis of potential and indicated readers of H2AQ105me across the rDNA locus as described in Fig 3 based on peptide pulldown. *n* = 3, biological replicates, error bars are standard error of the mean. Significance was tested using an unpaired, 2‐tailed *t*‐test.

**Figure EV3:**
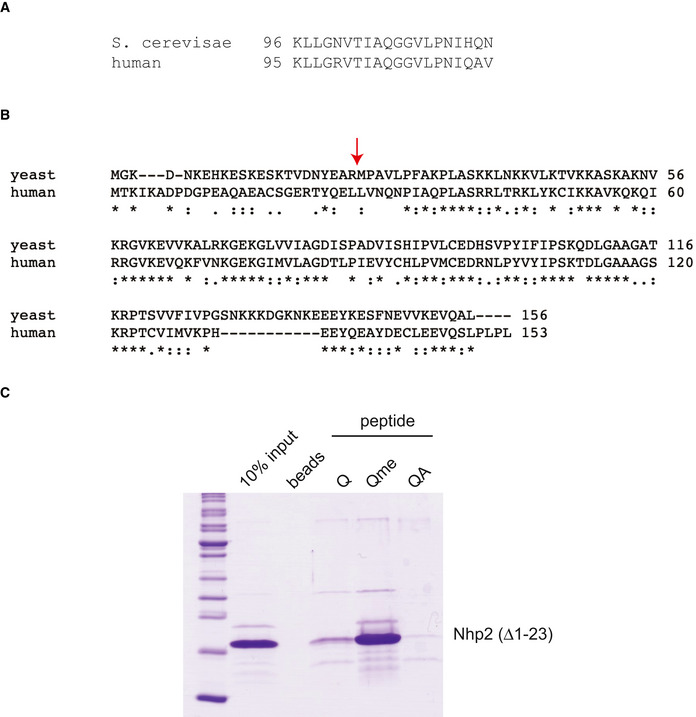
Conservation of H2AQ105me and Nhp2 Alignment of the region spanning yeast H2AQ105me comparing the *S*. *cerevisiae* and human sequence.Sequence Alignment between *S*. *cerevisiae* and human Nhp2 showing strong conservation with an exception of the N‐terminus and an insertion in yeast closer to the C‐terminus. Red arrow indicates new N‐terminus for an N‐terminally truncated version used in Fig EV3C.Peptide pulldowns using a N‐terminally (1‐23) truncated version of Nhp2 demonstrated that the less conserved N‐terminus is not involved in H2AQ105me recognition.

In order to gain further clarity on a possible mechanism for H2AQ105me‐mediated recruitment of ribosome biogenesis factors to the rDNA, it was important to identify a direct binder of glutamine methylation, an epigenetic reader of this histone modification. In order to do this, candidates were selected from the list of 139 Q105me‐enriched proteins identified by mass spec (Fig 2B), based on their known interactions listed on the yeast genome database (https://www.yeastgenome.org/). Initially, five potential readers reported to bind to H2A were myc‐tagged to allow for their easy detection and ChIPs were performed in wild type and H2AQ105A strains. Only one of these five candidates—Nhp2—was efficiently enriched at the rDNA in an H2AQ105me‐dependent manner (Figs 3A and EV2). However, recruitment of Nhp2 to the rDNA was not fully abolished in an H2AQ105A mutant, indicating that there are other ways to recruit Nhp2. Indeed, several high throughput assays have identified binding of Nhp2 to RNA Pol I itself (Fath *et al*, 2000; Lebaron *et al*, 2005; Krogan *et al*, 2006; Tarassov *et al*, 2008) as well as the rDNA chromatin component Hmo1 (Krogan *et al*, 2006). We then repeated the peptide pulldowns from yeast carrying the myc‐tagged Nhp2 allele. Nhp2 showed enrichment on the peptide harbouring the methylation on Q105, confirming the mass spectrometry data and highlighting Nhp2 as a possible epigenetic reader of H2AQ105me (Fig 3B). To confirm this hypothesis, Nhp2 was recombinantly purified from *Escherichia coli*. Peptide pulldowns using only recombinant Nhp2 were performed and analysed by SDS–PAGE and Coomassie staining (Fig 3C). This approach confirmed the ability of Nhp2 to directly bind to the peptide containing the methylated glutamine (Fig 3C). Furthermore, Nhp2 did not bind to methyl‐containing peptide sequences derived from histone H3, further confirming its specificity to methylation of Q105 on histone H2A (Fig 3C). The amino acids surrounding H2AQ105me are conserved from yeast to mammalian cells (Fig EV3A)—as is Nhp2, except for the N‐terminal region (Fig EV3B). However, the N‐terminus does not contribute to H2AQ105me binding (Fig EV3C). To test whether the Nhp2‐H2AQ105me interaction might also be evolutionarily conserved, we performed peptide pulldowns using mammalian cell lysates. While the interaction was not as strong as for yeast, human Nhp2 clearly interacts specifically with methylated H2AQ105 (Fig 3D), confirming the evolutionary conservation of the Nhp2‐H2AQ105me interaction. Finally, we wanted to address whether Nhp2 recruitment would follow H2AQ105 methylation genome wide. We performed ChIP‐sequencing using Nhp2‐myc. In line with the data described above, the ChIP profile of Nhp2 closely resembles the one of H2AQ105me with a strong enrichment over the rDNA region on yeast chromosome XII (Fig 3E), corroborating the idea that Nhp2 is an epigenetic reader of methylated H2AQ105. While the rDNA is the site for the strongest enrichment of Nhp2, it should be noted, that we identified further peaks across the genome (Dataset EV2). Genes associated with those peaks were associated mainly with nucleotide metabolic terms (Appendix Table S1).

### Nhp2 bridges H2AQ105me to ribosome biogenesis factors

**Figure 4:**
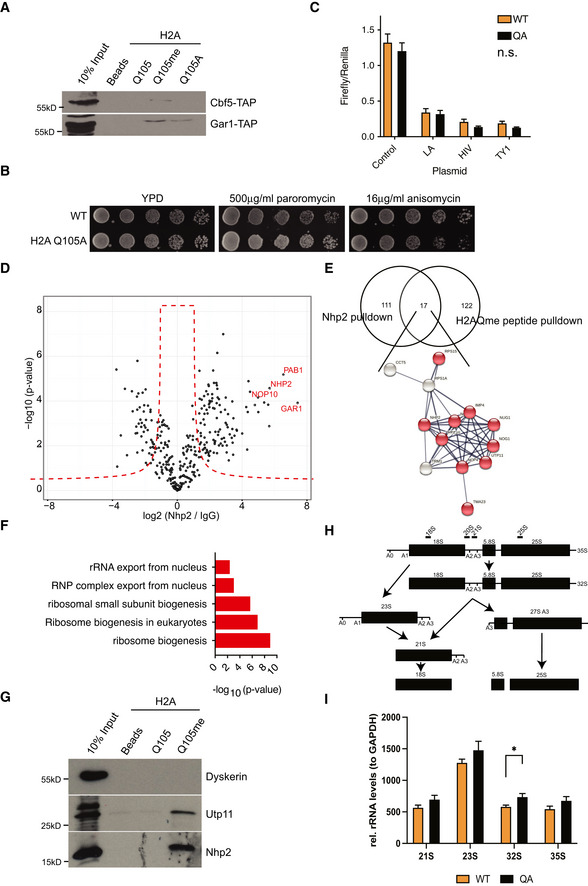
Nhp2 bridges components of the SSU and H2AQ105me Peptide pulldowns from indicated TAP‐tagged strains. Pulldown efficiency was analysed by Western blotting.Spot test of the indicated strains on YPD or YPD supplemented with the given antibiotics. Overnight yeast cultures were adjusted to OD_600_ = 1 and then serially diluted 5‐fold before spotting on indicated media.Ribosome slippage assay using a tandem luciferase reporter separated by a sequence allowing for slippage. The sequences allowing for slippage are given below the graph. *n* = 8, biological replicates, and error bars are standard error of the mean. Significance was tested using 2‐tailed, unpaired *t*‐test.Proteomic analysis of Nhp2 interactors. Highlighted in red are the known members of the H/ACA pseudouridylation complex. The red dashed line indicates significance cut‐off (FDR < 0.05; for a full list of enriched proteins, please refer to Dataset EV3).Intersection of Nhp2 and H2AQ105me pulldown. 17 proteins were enriched in both experiments and the majority are members of the small subunit processome (highlighted in red).GO enrichment for these 17 commonly identified proteins shown in Fig 4E.Peptide pulldowns from human cells (HEK293T) to test for conservation of the reported interactions between Nhp2 and members of the SSU processome complex on H2AQ105me. Dyskerin (Cbf5 homolog) was probed to test for the presence of members of the H/ACA complex. Pulldown efficiency was analysed by Western blotting using the indicated antibodies.Simplified schematic overview over some processing steps of the rRNA. Cut sites are indicated as A0‐A3. Blue bars indicate Northern probes to detect 18S and 25S mature rRNA, respectively (please also see Fig EV4). Labelled bars on top of the scheme indicate used probes. For a representative Northern blot, please be referred to the Source Data accompanying Fig 4.Quantification of Northern Blot results. Normalized to GAPDH, *n* = 3, error bars are standard error of the mean. Asterix indicates *P*‐value (*P < 0.05, based on unpaired, 2‐tailed *t*‐test). Source data are available online for this figure.

**Figure EV4:**
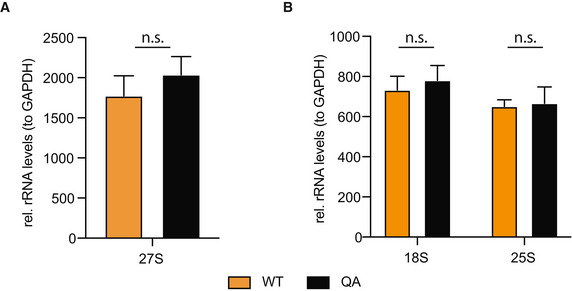
Levels of mature rRNA levels and 25S intermediates in an H2AQ105A mutant Levels of the 25S rRNA processing intermediate 27S show no difference in the level between a wild type (WT) and H2AQ105A (QA) mutant. *n* = 3, biological replicates, error bars are standard error of the mean. Significance was tested using an unpaired, 2‐tailed *t*‐test.Steady‐state levels of mature rRNA show no difference between a wild type (WT) and H2AQ105A (QA) mutant. *n* = 3, biological replicates, error bars are standard error of the mean. Significance was tested using an unpaired, 2‐tailed *t*‐test.

Nhp2 is a highly conserved protein that has been identified as a member of the H/ACA ribonucleoprotein complex and binds snoRNAs, of which we identified several to be de‐regulated in an H2AQ105A strain. Together with Gar1, Nop10 and the catalytic subunit, Cbf5, Nhp2 is responsible for the pseudouridylation of ribosomal RNA (Fath *et al*, 2000; Wang & Meier, 2004; Li *et al*, 2011). Nhp2 is an essential gene and is required for the maintenance of snoRNA levels (Henras *et al*, 1998). However, pseudouridylation of rRNA itself is not essential in yeast, but is required to maintain translational fidelity (Jack *et al*, 2011). Interestingly, pseudouridylation has been described to potentially occur co‐transcriptionally (Penzo & Montanaro, 2018). Yeast strains lacking pseudouridylation show an increase in frameshifts and are affected in tRNA binding to the ribosome (Jack *et al*, 2011). This can be easily measured by the sensitivity of yeast strains to several antibiotics that bind the A‐ and P‐sites of the ribosome. Strains lacking pseudouridylation are hyper‐sensitive to paromomycin and show higher resistance against anisomycin (Jack *et al*, 2011). While the hypothesis of H2AQ105me‐dependent recruitment of the pseudouridylation machinery was an attractive hypothesis, it is important to note that we did not detect an enrichment for any of the other components of the pseudouridylation complex using peptide pulldowns (Fig 2B and Dataset EV1). Additional pulldowns using TAP‐tagged components of this complex confirmed the mass spectrometry data (Fig 4A). Additionally, we did not observe any change in sensitivity towards paromomycin or anisomycin in H2AQ105A strains (Fig 4B), indicating that pseudouridylation is not strongly affected in the absence of H2AQ105‐methylation. To confirm this, we used ribosome translational slippage reporters as a more sensitive assay for changes in pseudouridylation status of the rRNA (Jack *et al*, 2011). In these reporters, the firefly and renilla luciferases are separated by sequences known to cause translational frameshift mutations. When this frameshift occurs, the translation of the second luciferase is induced (Harger & Dinman, 2003). No significant increase in translational frameshift was observed between wildtype and H2AQ105A strains (Fig 4C). Given that we were not able to identify evidence for a link between H2AQ105me and pseudouridylation, we considered that Nhp2 might have additional functions outside the H/ACA pseudouridylation complex. In order to determine the role that Nhp2 binding to H2AQ105me could play, we performed immunoprecipitation (IP) followed by quantitative mass spectrometry using the myc‐tagged version of Nhp2. Enrichment was calculated against the untagged parental strain. 128 proteins were found to be significantly, 2‐fold or more, enriched by myc in the Nhp2‐myc strain (Fig 4D and Dataset EV3). Among the top enriched proteins were all members of the pseudouridylation complex, indicating that the IP worked successfully (Fig 4D). To identify proteins that might be recruited to H2AQ105me via Nhp2, we intersected the list of Nhp2 binders with the proteins recruited to H2AQ105me (Fig 3B). 17 proteins were enriched in both experiments (Fig 4E). String analysis identified that 13 out of the 17 proteins formed a tight interaction network (Fig 4E). Gene ontology enrichment revealed these to be ribosome biogenesis factors, particularly involved in the processing of the SSU, such as Mpp10, Imp4 or Utp11 (Fig 4 E and F). Interestingly, all identified SSU components are involved in the early steps of rRNA maturation (Barandun *et al*, 2018). As the interaction between Nhp2 and H2AQ105me was conserved in humans (Fig 3D), we tested if the H2AQ105me‐based interaction between human Nhp2 and the SSU processome and the absence of H/ACA components might be conserved in humans as well. Therefore, we tested if Dyskerin (the human Cbf5 homolog) or Utp11 (member of the SSU processome) would interact with H2AQ105me. In line with the observations from yeast, Dyskerin was not pulled down on H2AQ105me‐containing peptides, while Utp11 was enriched to a similar extent as Nhp2 (Fig 4G), indicating that Nhp2 might indeed serve as an adapter protein involved in the recruitment of factors involved in the early steps of ribosome maturation. Finally, we wanted to test more directly if H2AQ105me might be involved in the early steps of ribosome processing. To this end, we performed Northern blot analysis using a probe to detect the first steps in 18S processing (32S, 23S and 21S – Fig 4H). Here, we observed a subtle, but significant enrichment of rRNA processing intermediates of the 18S rRNA (Fig 4I), while processing of the 25S rRNA (Fig EV4A) and the steady‐state levels of 18S and 25S rRNA were not affected (Fig EV4B). These results indicated that an H2AQ105A mutant has a mild rRNA processing defect and support the hypothesis that the H2AQ105me‐Nhp2 interaction might be required for optimal recruitment of SSU processome components. Interestingly, also mutating H3K56 to alanine leads to defects in SSU processome recruitment to the rDNA (Chen *et al*, 2012). As the SSU processome functions co‐transcriptionally (Gallagher *et al*, 2004; Phipps *et al*, 2011), it is likely that the Nhp2 recruitment by H2AQ105me, and the reported recruitment of the SSU processome via H3K56ac, allow for an efficient coupling of transcription and pre‐rRNA processing. Based on the data presented here, we propose a model by which the SSU processome is optimally recruited to the rDNA following the deposition of both H3K56ac and H2AQ105me and the subsequent recruitment of Nhp2.

## Materials and Methods

### Strains, plasmids and reagents

Genotypes of yeast strains and plasmids are listed in Appendix Tables S2 and S3. All chemicals used in this study were purchased analytical grade from either Sigma‐Aldrich or Carl Roth, unless stated otherwise. Drop Out Mix for yeast synthetic medium was from US Biological Life Sciences (D9543‐01). 5‐FOA was bought from Cayman Chemicals (17318). Protein‐G coupled Dynabeads™ (10004D) and streptavidin‐coated Dynabeads™ MyOne™ Streptavidin C1 (65001) were from Thermo Fisher. Secondary antibodies against rabbit (7074S) and mouse IgG (7076S) coupled to HRP were purchased from Cell Signaling. Primary antibodies were as follows: H3K56ac (Active Motif – 39281), G6PDH (Sigma – A9521) and c‐Myc (9e10, Sigma – MABE282), FLAG (Sigma – F3165), H2AQ105me from (Tessarz *et al*, 2014), H2A (Abcam – ab13923), H3 (Abcam – ab1791), Utp11L (GeneTex GTX115929), Dyskerin (Santa Cruz sc‐373956) and human Nhp2 (Proteintech 15128‐1‐AP). ECL solution was from Promega (W1001). T4 Polynucleotide kinase was from NEB (M0201) and Probe Quant G‐50 microcolumns from GE Healthcare (28‐9034‐08). γ‐(^32^P)‐ATP was purchased from Perkin Elmer (BLU002Z250UC).

### Yeast protocols

If not stated otherwise, all strains used were derived from W303. Gene deletions or tag integrations were performed using PCR‐based methods (Longtine *et al*, 1998; Janke *et al*, 2004). Yeast were grown in YPD medium if not stated differently. Histone mutants were generated using plasmid shuffling. For spot tests, cells were grown overnight to stationary phase in YPD, diluted to an optical density (OD_600nm_) of 1 and serially diluted 5‐fold before spotting on YPD plates containing the relevant antibiotics.

### Protein precipitation, SDS–PAGE and Western blot

Protein was extracted from a yeast pellet of OD_600nm_ of 1, using sodium hydroxide lysis and TCA precipitation as previously described (Knop *et al*, 1999). Precipitated proteins were dissolved in 50 µl of 2× Laemmli loading buffer and boiled for 10 min. 10 µl was loaded per well of a 15% SDS–PAGE, which was run at 120–200 V in 1× SDS gel running buffer until the bromophenol blue marker reached the bottom of the gel. Separated proteins were either visualized directly using coomassie blue staining or transferred to a nitrocellulose membrane using 1× carbonate buffer (10 mM NaHCO_3_, 3 mM Na_2_CO_3_, pH 9.0, 20% methanol) at 400 mA for 75 min. Membranes were blocked with 5% BSA (for histone and histone modification antibodies) or 5% milk (for all other antibodies) in 1× TBST for one hour at room temperature. Primary antibody was added, following manufacturer’s recommended concentration, in blocking solution. Membranes were incubated overnight at 4°C with gentle rocking, subsequently incubated with secondary antibody and developed using ECL.

### Recombinant expression of Nhp2

pET24a‐Nhp2 was transformed into BL21 DE3 Codon Plus and plated on LB‐Agar, supplemented with kanamycin and chloramphenicol. Overnight cultures were set up and diluted to OD_600nm_ 0.1 in 3 × 1.5 l in 2YT supplemented with kanamycin and chloramphenicol and grown to OD_600nm_ 0.6 at 37°C. 2 mM IPTG were added for 3 h. Cells were harvested and 1 pellet per 1.5 l was frozen and stored at −80°C. For purification, each pellet was resuspended in 35 ml WB 1 (20 mM Tris pH 7.5, 300 mM NaCl, 5 mM β‐Mercaptoethanol, 10 mM Imidazole) and sonicated on ice using a Branson sonifier 450 with 50% duty cycle and output control 2 for 6 × 30 s with 1 min incubation on ice in between. Cell lysates were centrifuged at 20,000 *g* for 30 min at 4°C. Lysates were combined, passed through a 0.45 μm filter and incubated with 1 ml Ni‐NTA beads (Qiagen) for 1 h at 4°C. Beads were washed with 50 ml WB1, followed by 50 ml WB2 (20 mM Tris pH 7.5, 100 mM NaCl, 5 mM β‐Mercaptoethanol, 10 mM Imidazole) and eluted in 1 ml fractions using EB1 (20 mM Tris pH 7.5, 100 mM NaCl, 5 mM β‐Mercaptoethanol, 250 mM Imidazole). Fractions were checked by SDS–PAGE for protein and those containing Nhp2 were pooled and passed over a 2 ml SP‐sepharose column equilibrated with SP1 (20 mM Tris pH 7.5, 100 mM NaCl, 5 mM β‐Mercaptoethanol). Column was washed with 50 ml SP1, and Nhp2 was eluted with SP2 (20 mM Tris pH 7.5, 1 M NaCl, 5 mM β‐Mercaptoethanol) and fractions of 1 ml were collected and checked for Nhp2. Fractions containing pure Nhp2 were pooled and dialyzed against storage buffer (20 mM Tris pH 7.5, 150 mM NaCl, 5 mM β‐Mercaptoethanol, 10% glycerol), aliquoted, snap frozen and stored at −80°C.

### Chromatin immunoprecipitation, qPCR or next‐generation sequencing and analysis

Yeast was grown into mid‐log phase, (OD_600nm_ 0.6) in YPD, and 50 ml was harvested and placed into 50 ml falcon tubes. 1% formaldehyde (final concentration) was added, and cultures were left to rotate at room temperature for 30 min to allow for crosslinking. Cultures were centrifuged (3 K, 2 min, 4°C), and supernatant was discarded. Cell pellets were resuspended in 20 ml cold PBS, centrifuged as above and the supernatant discarded again. This washing was repeated 2 times in total, and cells were then transferred to 2‐ml Eppendorf tubes and kept on ice. Pellets were then resuspended in 500 µl SDS buffer (1% SDS, 10 mM EDTA, 50 mM Tris‐Cl (pH 8.0), plus protease inhibitors) on ice. About 200 µl of acid‐washed glass beads were added and tubes were vortexed at top speed at room temperature for 6 × 1 min, with 3 min on ice in between. The bottoms of the tubes were pierced with a hot needle and place in 15 ml falcon tubes, which were centrifuged (2k, 30 s) to release the cell lysate. Lysates were sonicated to produce fragments of 200–600 bp. Sonicated samples were transferred to 1.5 ml Eppendorf tubes, centrifuged (20 min, 14 K, 4°C) and diluted in 5 ml of cold IP buffer (0.01% SDS, 1.1% Triton X‐100, 1.2 mM EDTA, 16.7 MM Tris‐Cl (pH 8.0), 167 mM NaCl, plus protease inhibitors). 50 µl was taken as input control and stored at −2°C. For the IP, 1 ml of chromatin was placed in siliconized Eppendorf tubes and 2 µg of antibody was added. Chromatin/antibody mix was place on rotating wheel at 4°C overnight. 30 µl of Protein G Dynabeads™ was washed 3× in IP buffer and added to the chromatin/antibody mix. This was incubated at room temperature on a rotating wheel for 90 min. Beads were washed for 3 min with each wash buffer (1 ml TSE (1% Triton X‐100, 0.1%SDS, 2 mM EDTA, 20 mM Tris‐Cl (pH 8.0)) plus 150 mM NaCl, 1 ml TSE plus 500 mM NaCl, 1 ml LiCl wash (0.25 M LiCl, 1% NP‐40, 1% deoxycholate, 1 mM EDTA, 10 mM Tris‐Cl (pH 8.0)) and 1 ml TE (pH8.0)). IP was eluted using 200 µl of Elution buffer (1% SDS, 0.1 M NaHCO_3_), made fresh on the day, on a rotating wheel for 30 min at room temperature. 20 µl of 5 M NaCl was added to all samples, which were then incubated at 95°C for 15 min, or overnight at 65°C, to reverse the formaldehyde crosslink. 5 µg of DNase‐free RNase was then added for 30 min at 37°C, and then, the DNA was purified using a Qiagen PCR purification kit. 1 µl was analysed per reaction by qPCR. ChIP‐seq libraries were generated from two independent biological replicates of IgG, Nhp2‐myc and input following a previously published protocol (Ford *et al*, 2014) and sequenced on an Illumina HighSeq 4000 using 1 × 75 bp reads. A detailed version of this protocol is available upon request.

### RNA‐seq analysis

Total RNA was isolated using the hot phenol. RNA quality was analysed using an Agilent Tapestation and only RNA with RIN > 9 was used for RNA‐seq library production. Libraries were generated at the Genomics Core of the Max Planck Institute for Plant Breeding Research, Cologne Germany using the NEBNext^®^ Ultra™ RNA Library Prep Kit on rRNA‐depleted total RNA. Sequencing reads were mapped to yeast genome (R64) using bowtie2 (Langmead & Salzberg, 2012). Gene counts were obtained from featureCounts of the Rsubread package (R/Bioconductor) (Liao *et al*, 2013). Gene set enrichment analysis was performed using R package fgsea (preprint: Korotkevich *et al*, 2021) using the *S*. *cerevisiae* GSEA.gmt file provided at http://ge‐lab.org/gskb/ (downloaded May 2021).

### ChIP‐seq analysis

ChIP‐seq data were mapped to the yeast genome (R64) using bowtie2 (Langmead & Salzberg, 2012). Mapped reads were indexed and merged using samtools (Li *et al*, 2009) and converted to bigwig files using deepTools bamCoverage (v 2.0; Ramírez *et al*, 2016) with a bin size of 10 and normalization to genomic content. Single gene tracks were generated through the IGV genome browser. Peak calling was performed using Macs2 (Zhang *et al*, 2008) with peaks displaying an FDR < 10^−5^ considered statistically significant. The H2AQ105me track was directly taken from Tessarz *et al*, 2014 (E‐MTAB‐1447) without any further analysis.

### SILAC labelling

BY4742 was grown in 1 l of SD supplemented with amino acids and uracil, excluding lysine. Cultures were then supplemented with 100 mg/l of either normal lysine or heavy lysine (Sigma, L‐Lysine‐^13^C_6_^15^N_2_ hydrochloride cat. number 608041) and grown for no < 15 h to reach an OD_600nm_ of 0.6–0.8, to allow for maximum labelling of proteins. Cells were then harvested following the peptide‐pulldown protocol.

### Yeast lysates

Cells were grown in 200 ml of YPD (for SILAC labelling see specific culture protocol) and harvested and washed 1× in 1× PBS, to get rid of excess media, and resuspended in a high‐salt binding buffer (50 mM Tris‐Cl (pH 8.0), 1% NP40, 420 mM NaCl, 1 mM DTT and protease inhibitors) 1:2 (yeast:buffer). Yeast suspension was dropped directly into liquid nitrogen to snap freeze and then lysed using a Freezer Mill (Brand). Cell debris was removed by centrifugation (10 K, 4**°**C, 2 min), and the supernatant was transferred to a clean tube and diluted 1:1 with no‐salt binding buffer (50 mM Tris‐Cl (pH 8.0), 1% NP40, 1 mM DTT and protease inhibitors). Protein content was verified by Bradford assay, and the lysate was further diluted, in binding buffer (50 mM Tris‐Cl (pH 8.0), 1% NP40, 150 mM NaCl, 1 mM DTT and protease inhibitors), to obtain a concentration of about 1.7 mg/ml.

### Peptide pull‐downs

600 μl of cleared yeast lysates or lysates from HEK293T (about 1 mg of protein) was used per pulldown. For peptide pulldowns, cell lysates were incubated with peptide‐coupled magnetic beads Dynabeads ^TM^ MyOne ^TM^ Streptavidin C1. Lysate/bead mix was rotated at 4**°**C for 2 h and then washed 5× in 1 ml of binding buffer. For analysis by Western blot, proteins were eluted in 2× Laemmli buffer for 10 min at 65**°**C, boiled for a further 10 min and then loaded onto an SDS–PAGE and analysed by Western blot. For mass spectrometry, see Mass Spectrometry section. Beads were washed 5× with 1 ml TBS at 4**°**C before protein elution in mass spec elution buffer. For the Nhp2‐myc IP, 5 μl anti‐myc antibody was bound to 50 μl ProteinG Dynabeads for at least 2 h at 4**°**C in TBS. Cleared protein lysates were adjusted to 1 mg/ml, and 1 ml was added to prebound antibodies and incubated at 4**°**C overnight. Beads were washed 5× with 1 ml TBS at 4**°**C before protein elution in mass spec elution buffer (see Mass Spectrometry protocol).

### Mass spectrometry sample preparation

Following peptide pulldown of SILAC‐labelled cultures, beads from heavy and light experiments were mixed and washed further 2× in 1 ml of 1× TBS to remove excess detergent. Proteins were eluted in 50 ul of mass spec elution buffer (6 M Guanidine hydrochloride, 10 mM TCEP, 40 mM CAA, 100 mM Tris (pH 8.5)), for 1 h at room temperature, regularly shaking to prevent the beads from settling and then diluted 10‐fold in 20 mM Tris/10% acetronile. The protein concentration was measured using a nanodrop (260/280), and 5 ug of LysC was added to 100 ug of protein. Proteins were digested on the beads at 37**°**C overnight with gentle shaking so as to prevent the beads from settling. Following protein digestion, peptides were purified using FASP peptide purification (Coleman *et al*, 2017), followed by Mass spec analysis. The analysis was performed on five biological replicates using forward and reverse pulldowns, i.e. that heavily‐labelled cultures were once incubated with the methylated peptide (forward) and in a second experiment with the unmethylated (reverse). Unmethylated peptide was incubated with the respective other peptide.

### LC‐MS/MS analysis

Peptides were separated on a 25 cm, 75 μm internal diameter PicoFrit analytical column (New Objective) packed with 1.9 μm ReproSil‐Pur 120 C18‐AQ media (Dr. Maisch,) using an EASY‐nLC 1000 (Thermo Fisher Scientific). The column was maintained at 50°C. Buffer A and B were 0.1% formic acid in water and 0.1% formic acid in acetonitrile. For the Q105me (Qme) IP, peptides were separated on a segmented gradient from 5% to 20% buffer B for 100 min, from 20% to 25% buffer B for 10 min and from 25% to 40% buffer B for 10 min at 200 nl/min. For the Nhp2 IP, peptides were separated on a segmented gradient from 6% to 31% buffer B for 45 min and from 31% to 44% buffer B for 8 min at 200 nl/min. Eluting peptides were analysed on a QExactive Plus (Qme IP) or QExactive HF (Nhp2 IP) mass spectrometer (Thermo Fisher Scientific). Peptide precursor m/z measurements were carried out at 70,000 (Qme IP) or 60,000 (Nhp2 IP) resolution in the 300–1,800 (Qme IP) or 300–1,500 (Nhp2 IP) m/z range. The top ten most intense precursors with charge state from 2 to 7 only were selected for HCD fragmentation using 25% (Qme IP) or 27% (Nhp2 IP) normalized collision energy. The m/z values of the peptide fragments were measured at a resolution of 17,500 (Qme IP) or 15,000 (Nhp2 IP) using 80 ms maximum injection time. Upon fragmentation, precursors were put on a dynamic exclusion list for 45 s.

### Protein identification and quantification

The raw data were analysed with MaxQuant version 1.5.2.8 (Cox & Mann, 2008) using the integrated Andromeda search engine (Cox *et al*, 2011). Peptide fragmentation spectra were searched against the canonical and isoform sequences of the yeast reference proteome (proteome ID UP000002311, downloaded February 2015 from UniProt). Methionine oxidation and protein N‐terminal acetylation were set as variable modifications; cysteine carbamidomethylation was set as fixed modification. The digestion parameters were set to “Specific” and “LysC/P” (Qme IP) or “Trypsin/P” (Nhp2 IP). The minimum number of peptides and razor peptides for protein identification was 1; the minimum number of unique peptides was 0. Protein identification was performed at a peptide spectrum matches and protein false discovery rate of 0.01. The “second peptide” option was on. For the analysis of the Qme IP, “Re‐quantify” was enabled. For the analysis of the Nhp2 IP, successful identifications were transferred between the different raw files using the “Match between runs” option. SILAC quantification (Qme IP) was performed using a minimum ratio count of two. Label‐free quantification (Nhp2 IP) (Cox *et al*, 2014) was performed using a minimum ratio count of two. Downstream data transformation, filtering and differential abundance analysis was performed using Perseus version 1.5.0.0 (Tyanova *et al*, 2016). For the Qme IP data, log_2_ transformed SILAC ratios were analysed using a one‐sided *t*‐test against zero. For the Nhp2 IP data, label‐free intensities were filtered for at least three valid values in at least one group and imputed from a normal distribution with a width of 0.3 and down shift of 1.8. Imputed values were analysed with a two‐sided *t*‐test, using a S0 parameter of one.

### Northern blotting

Total RNA of logarithmically growing yeast (PTY1126 and PTY1132) was isolated using the hot phenol approach, and 10 µg of total RNA per lane was run on 1.2% agarose gels to separate high molecular weight RNA samples at 0.5 V/cm for 24 h at 4°C. Following a rinse in dH_2_O, the gel was then washed 2 × 15 min in Tris/Salt buffer (1.5 M NaCL, 0.5 M Tris, 30 ml HCl), quickly rinsed again in dH_2_O and then soaked for 20 min in 6× SSC (0.9 M NaCl, 90 mM Na3 citrate, pH 7.0). The transfer stack was then set up at room temperature and transfer, per capillary action, of the RNA to a nylon membrane (Amersham Hybond‐N+, GE Healthcare) was allowed to take place overnight (16–24 h) in 6× SSC. Following transfer, the RNA was immobilized by UV crosslinking (80 mJ/cm^2^). The membrane was prehybridized for 1 h at 37°C in 10× Denhardt hybridization buffer (100× Denhardt = 2% Ficoll 400, 300 mM NaCl, 2% polyvinylpyrrolidone, 2% BSA). Oligonucleotide probes (18S: 5’‐CATGGCTTAATCTTTGAGAC‐3’, 25S: 5’‐ CTCCGCTTATTGATATGC‐3’, 21S: 5’‐ GATATGAAAACTCCACAGTG‐3’ and GAPDH: 5’‐ CGGTTCTACCACCTCTCCAG‐3’) were labelled with γ‐(^32^P)‐ATP using T4 Polynucleotide kinase and purified using Probe Quant G‐50 microcolumns. 1 mM probe was used to hybridize to the RNA overnight. After exposing the radioactively labelled membrane to a phosphor screen (GE health care) for at least 24 h, the screen was imaged using a Fuji FLA‐7000 laser scanner. All Northern blot experiments were done in three biological repeats. Bands were quantified using the Multi Gauge V3.2 software. The photostimulated luminescence (PSL) value was used as a representation of abundance of RNA. GAPDH was used as a loading control. The relative concentration for each individual sample was determined.

## Author contribution

JSPM and PT designed the study; JSPM, JM, CR, NG and PT generated and analysed data; JM and CM analysed ChIP‐ and RNA‐seq data; JSPM and PT wrote the manuscript with input from all authors.

## Conflict of interest

The authors declare that they have no conflict of interest.

## Supporting information



Appendix

Expanded View Figures PDF

Dataset EV1

Dataset EV2

Dataset EV3

Source Data for Figure 1

Source Data for Figure 3

Source Data for Figure 4

## Data Availability

The mass spectrometry proteomics data have been deposited to the ProteomeXchange Consortium via the PRIDE (Perez‐Riverol *et al*, 2019) partner repository with the dataset identifier PXD023510 (http://www.ebi.ac.uk/pride/archive/projects/PXD023510). Analysed data can be found in the Appendix. RNA‐ and ChIP‐seq data are available at gene expression omnibus, accession number: GSE176302 (https://www.ncbi.nlm.nih.gov/geo/query/acc.cgi?acc=GSE176302).
