# Supplementary material for: Nhp2 is a reader of H2AQ105me and part of a network integrating metabolism with rRNA synthesis
Source: EMBO Rep. 2021 Aug 19;22(10):e52435. doi: 10.15252/embr.202152435 (PMC8490984; doi:10.15252/embr.202152435)
Supplement: Supplementary file 1 — Appendix [file EMBR-22-e52435-s001.pdf]

Appendix

Appendix Table S1: GO term enrichment for genes associated with Nhp2 peaks *(p. 1)*

Appendix Table S2: Yeast strains used in this study *(p. 2)*

Appendix Table S3: Plasmids used in this study *(p. 3)*

Appendix Table S1

GOID	GO_term	P-value	FDR	Expected FP
46031	ADP metabolic process	0.00360	0.00	0.00
6090	pyruvate metabolic process	0.00383	0.00	0.00
9135	purine nucleoside diphosphate metabolic process	0.00488	0.00	0.00
9179	purine ribonucleoside diphosphate metabolic process	0.00488	0.00	0.00
9185	ribonucleoside diphosphate metabolic process	0.00488	0.00	0.00
9132	nucleoside diphosphate metabolic process	0.00864	0.00	0.00

Appendix Table S2

Strain	genotype	source
PTY1053	Mata ade2-1 ura3-1 his3-11,15 t rp1-1 leu2-3,112 can1-100 hta1-htb1::kanMX4 hta2-htb2::natMX4	Tessarz et al., 2014
PTY1055	Mata ade2-1 ura3-1 his3-11,15 trp1-1 leu2-3,112 can1-100 hta1-htb1::kanMX4 hta2-htb2::natMX4 p(HIS3)-HTA1-HTB1	Tessarz et al., 2014
PTY1056	Mata ade2-1 ura3-1 his3-11,15 trp1-1 leu2-3,112 can1-100 hta1-htb1::kanMX4 hta2-htb2::natMX4 p(HIS3)-HTA1Q105A-HTB1	Tessarz et al., 2014
PTY1021	ade2-1 ura3-1 his3-11,15 trp1-1 leu2-3,112 can1-100 hht1-hhf1::kanMX4 hht2-hhf2::natMX4 p(URA3)-HHT2/HHF2	this study
PTY1035	Mata ade2-1 ura3-1 his3-11,15 trp1-1 leu2-3,112 can1-100 asf1::kanMX4	this study
PTY1036	Mata ade2-1 ura3-1 his3-11,15 trp1-1 leu2-3,112 can1-100 rtt109::kanMX4	this study
PTY1040	Mata ade2-1 ura3-1 his3-11,15 trp1-1 leu2-3,112 can1-100 cac1::kanMX4	this study
PTY1042	Mata ade2-1 ura3-1 his3-11,15 trp1-1 leu2-3,112 can1-100 hst3::kanMX4	this study
PTY1043	Mata ade2-1 ura3-1 his3-11,15 trp1-1 leu2-3,112 can1-100 hst4::kanMX4	this study
PTY1051	Mata ade2-1 ura3-1 his3-11,15 trp1-1 leu2-3,112 can1-100 hht1-hhf1::kanMX4 hht2-hhf2::natMX4 rtt109::hphMX4 p(TRP1)-HHT2-HHF2	this study
PTY1052	Mata ade2-1 ura3-1 his3-11,15 trp1-1 leu2-3,112 can1-100 hht1-hhf1::kanMX4 hht2-hhf2::natMX4 rtt109::hphMX4 p(TRP1)-HHT2K56A-HHF2	this study
PTY1136	Nhp2-3Myc-TRP1 Mata ade2-1 ura3-1 his3-11,15 trp1-1 leu2-3,112 can1-100 hta1-htb1::kanMX4 hta2-htb2::natMX4 p(URA)-HTA1-HTB1	this study
Y7092	MATα can1::STE2pr-Sp_his5 lyp1Δ his31Δ leu2Δ0 ura3Δ0 met17Δ0 LYS2+	Tong et al., 2001
Y8205	can1Δ ::STE2pr-Sp_his5 lyp1Δ ::STE3pr-LEU2 his3Δ1 leu2Δ0 ura3Δ0	Tong et al., 2001
Y8205.X	can1Δ ::STE2pr-Sp_his5 lyp1Δ ::STE3pr-LEU2 his3Δ1 leu2Δ0 ura3Δ0 H2AQ105A::URA3	this study
PTY1136	Nhp2-3Myc-TRP1 Mata ade2-1 ura3-1 his3-11,15 trp1-1 leu2-3,112 can1-100 hta1-htb1::kanMX4 hta2-htb2::natMX4	this study
GAR1-TAP	MATa his3Δ1 leu2Δ0 met15Δ0 ura3Δ0 GAR1-TAP::HIS3MX6	TAP-tag collection
CBF5-TAP	MATa his3Δ1 leu2Δ0 met15Δ0 ura3Δ0 CBF5-TAP::HISMX6	TAP-tag collection
PTY1386	Mata ade2-1 ura3-1 his3-11,15 trp1-1 leu2-3,112 can1-100 rpl9::hphNT1 hta1-htb1::kanMX4 hta2-htb2::natMX4 p(URA)-HTA1-HTB1	this study
PTY1387	Mata ade2-1 ura3-1 his3-11,15 trp1-1 leu2-3,112 can1-100 rpl1a::hphNT1 hta1-htb1::kanMX4 hta2-htb2::natMX4 p(URA)-HTA1-HTB1	this study
PTY1388	Mata ade2-1 ura3-1 his3-11,15 trp1-1 leu2-3,112 can1-100 rpl12a::hphNT1 hta1-htb1::kanMX4 hta2-htb2::natMX4 p(URA)-HTA1-HTB1	this study

Appendix Table S3

plasmids	description	Reference
pET24a-Nhp2	overexpression vector for Nhp2, kann selection, T7 regulation	this study
p(TRP1)-HHT2-HHF2	shuffle plasmid to introduce WT H3	this study
p(TRP1)-HHT2S10A-HHF2	shuffle plasmid to introduce H3S10A	this study
p(TRP1)-HHT2K56A-HHF2	shuffle plasmid to introduce H3K56A	this study
p(TRP1)-HHT2-HHF2K5A	shuffle plasmid to introduce H4K5A	this study
p(TRP1)-HHT2-HHF2K12A	shuffle plasmid to introduce H4K12A	this study
p(TRP1)-HHT2-HHF2KK5,12AA	shuffle plasmid to introduce H4KK5,12AA	this study
p(HIS3)-HTA1-HTB1	shuffle plasmid to introduce WT H2A	Tessarz et al., 2014
p(HIS3)-HTA1Q105A-HTB1	shuffle plasmid to introduce H2AQ105A	Tessarz et al., 2014
pRS314-Nop1-FLAG	NHP2 cds +/- 500bp; 3xmyc inserted at C-terminus	this study
pJD187.WT	slippage reporter, CTRL	Harger and Dinman, 2003
pJD376	slippage reporter, LA	Harger and Dinman, 2003
pJD377	slippage reporter, Ty1	Harger and Dinman, 2003
pJD378	slippage reporter, HIV	Harger and Dinman, 2003
