# Supplementary material for: Nhp2 is a reader of H2AQ105me and part of a network integrating metabolism with rRNA synthesis
Source: EMBO Rep. 2021 Aug 19;22(10):e52435. doi: 10.15252/embr.202152435 (PMC8490984; doi:10.15252/embr.202152435)
Supplement: Supplementary file 2 — Expanded View Figures PDF [file EMBR-22-e52435-s005.pdf]

Expanded View Figures

A

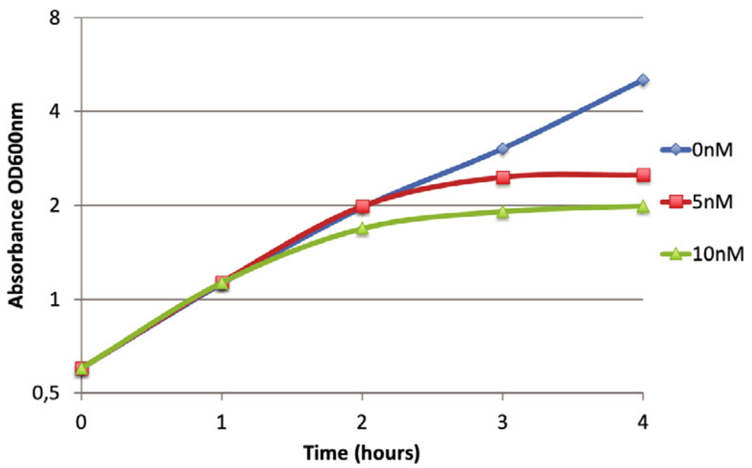

B

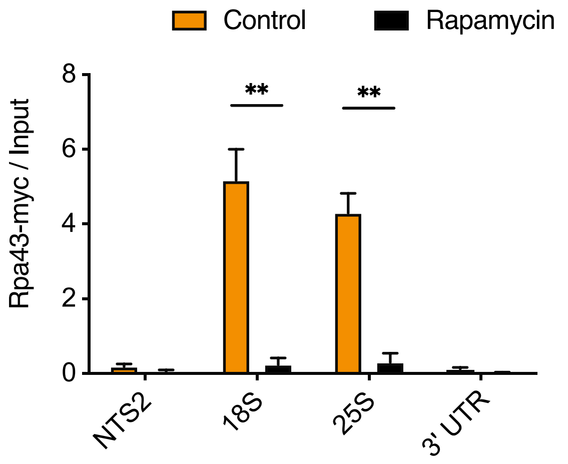

Figure EV1. Rapamycin reduces RNA Pol I transcription of rDNA.

A Growth curve of WT yeast cells challenged with indicated concentrations of rapamycin.

B Loss of RNA Pol I occupancy at the rDNA upon treatment with 5 nM rapamycin. $n = 3$, biological replicates, error bars are standard error of the mean. $^{**}P < 0.001$, based on unpaired, 2-tailed *t*-test.

 © 2021 The Authors

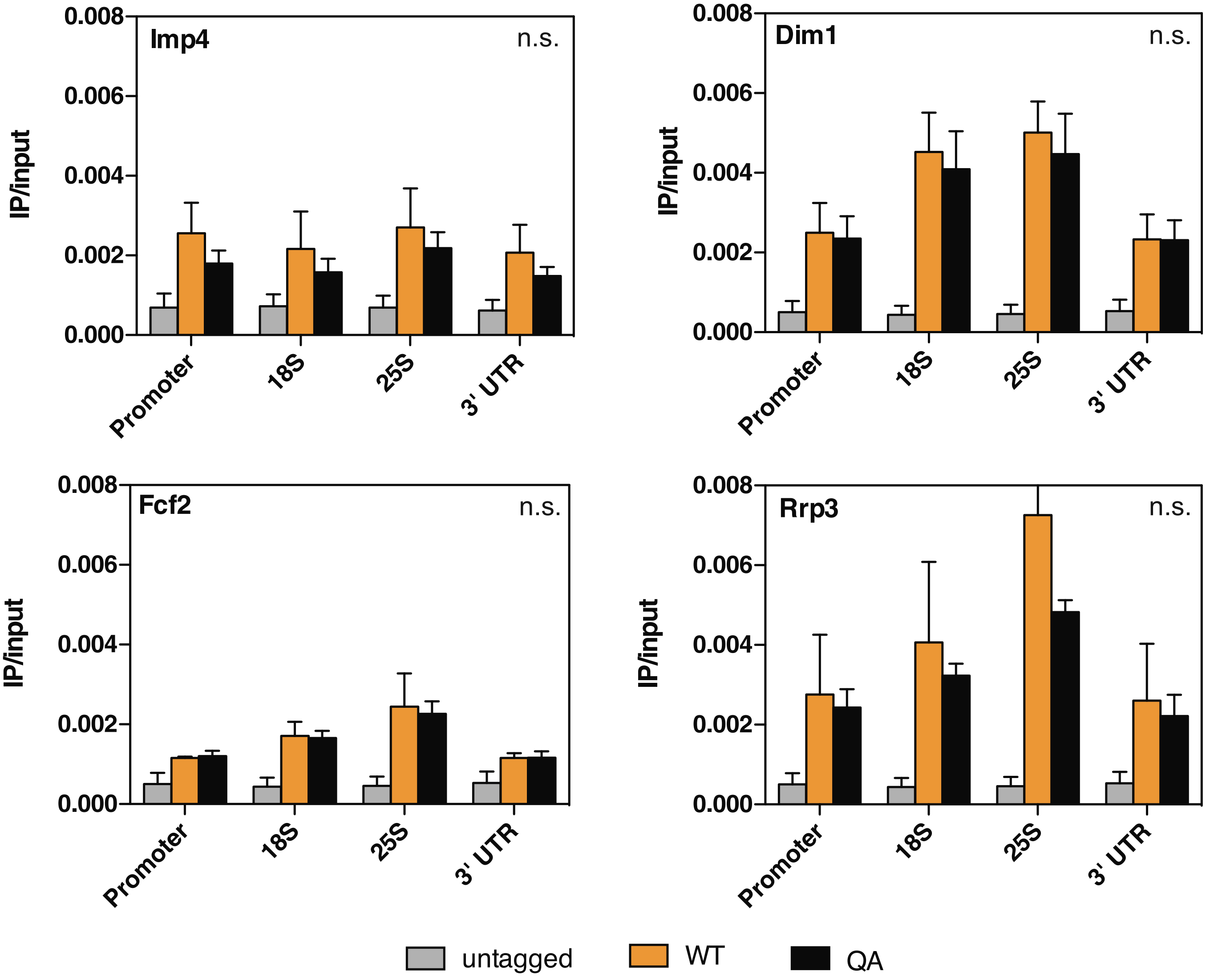

Figure EV2. ChIP-qPCR analysis of potential H2AQ105me readers.

ChIP-qPCR analysis of potential and indicated readers of H2AQ105me across the rDNA locus as described in Fig 3 based on peptide pulldown. $n = 3$, biological replicates, error bars are standard error of the mean. Significance was tested using an unpaired, 2-tailed t-test.

© 2021 The Authors

A

```
S. cerevisae    96 KLLGNVTIAQGGVLPNIHQN
human           95 KLLGRVTIAQGGVLPNIQAV
```

B

```
yeast     MGK---D-NKEHKESKESKTVDNYEARMPAVLPFAKPLASKKLNKKVLKTVKKASKAKNV 56
human     MTKIKADPDGPEAQAEACSGERTYQELLVNQNPIAQPLASRRLTRKLYKCIKKAVKQKQI 60
          * *   * :  . ::: ..   .*:  :    *:*:****::*.:*: * :*** * *::

yeast     KRGVKEVVKALRKGEKGLVVIAGDISPADVISHIPVLCEDHSVPYIFIPSKQDLGAAGAT 116
human     RRGVKEVQKFVNKGEKGIMVLAGDTLPIEVYCHLPVMCEDRNLPYVYIPSKTDLGAAAGS 120
          :****** * :.*****::*:***  * :* .*:**:***:.:**::**** *****..:

yeast     KRPTSVVFIVPGSNKKKDGKNKEEEYKESFNEVVKEVQAL---- 156
human     KRPTCVIMVKPH-----------EEYQEAYDECLEEVQSLPLPL 153
          ****.*::: *            ***:*:::* ::***:*
```

C

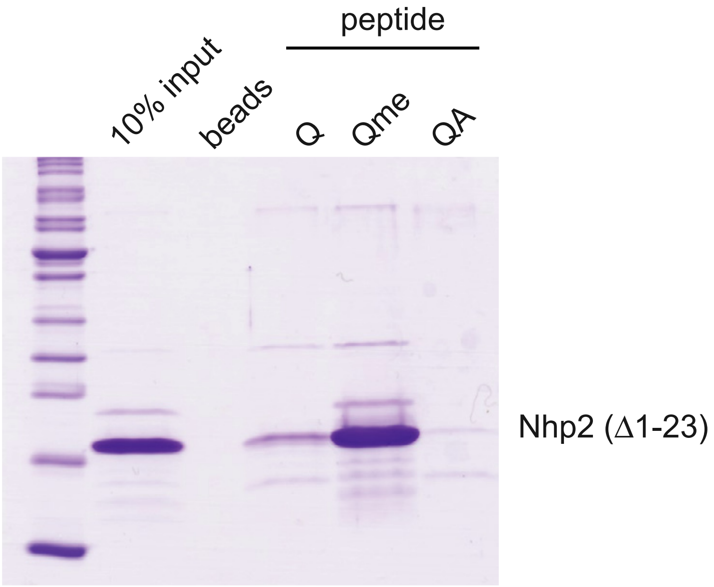

Figure EV3. Conservation of H2AQ105me and Nhp2.

A Alignment of the region spanning yeast H2AQ105me comparing the *S. cerevisiae* and human sequence.

B Sequence Alignment between *S. cerevisiae* and human Nhp2 showing strong conservation with an exception of the N-terminus and an insertion in yeast closer to the C-terminus. Red arrow indicates new N-terminus for an N-terminally truncated version used in Fig EV3C.

C Peptide pulldowns using a N-terminally (1-23) truncated version of Nhp2 demonstrated that the less conserved N-terminus is not involved in H2AQ105me recognition.

 © 2021 The Authors

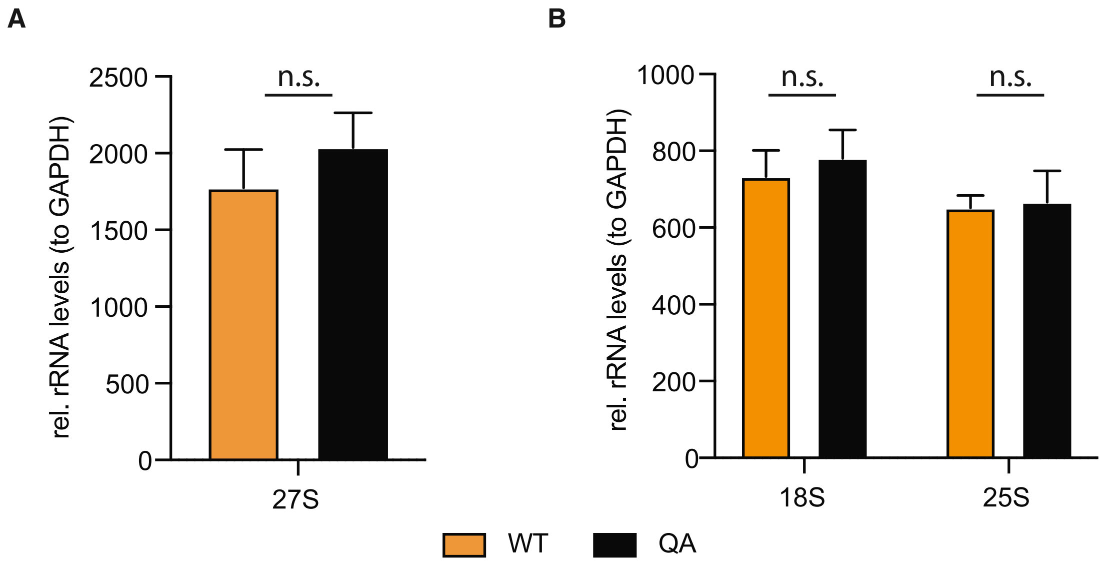

Figure EV4. Levels of mature rRNA levels and 25S intermediates in an H2AQ105A mutant.

A Levels of the 25S rRNA processing intermediate 27S show no difference in the level between a wild type (WT) and H2AQ105A (QA) mutant. n = 3, biological replicates, error bars are standard error of the mean. Significance was tested using an unpaired, 2-tailed t-test.

B Steady-state levels of mature rRNA show no difference between a wild type (WT) and H2AQ105A (QA) mutant. n = 3, biological replicates, error bars are standard error of the mean. Significance was tested using an unpaired, 2-tailed t-test.

© 2021 The Authors
