# Supplementary figures and images for: Nhp2 is a reader of H2AQ105me and part of a network integrating metabolism with rRNA synthesis
Source: EMBO Rep. 2021 Aug 19;22(10):e52435. doi: 10.15252/embr.202152435 (PMC8490984; doi:10.15252/embr.202152435)

Figure 1A

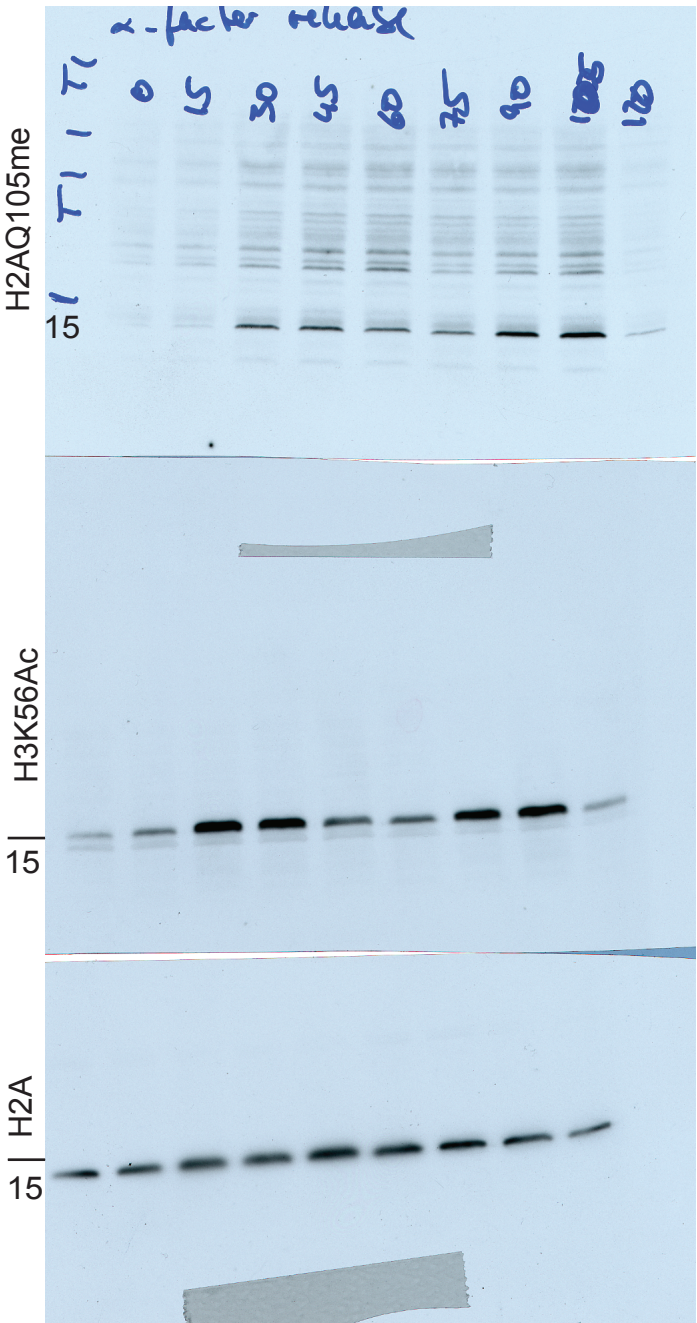

Figure 1B

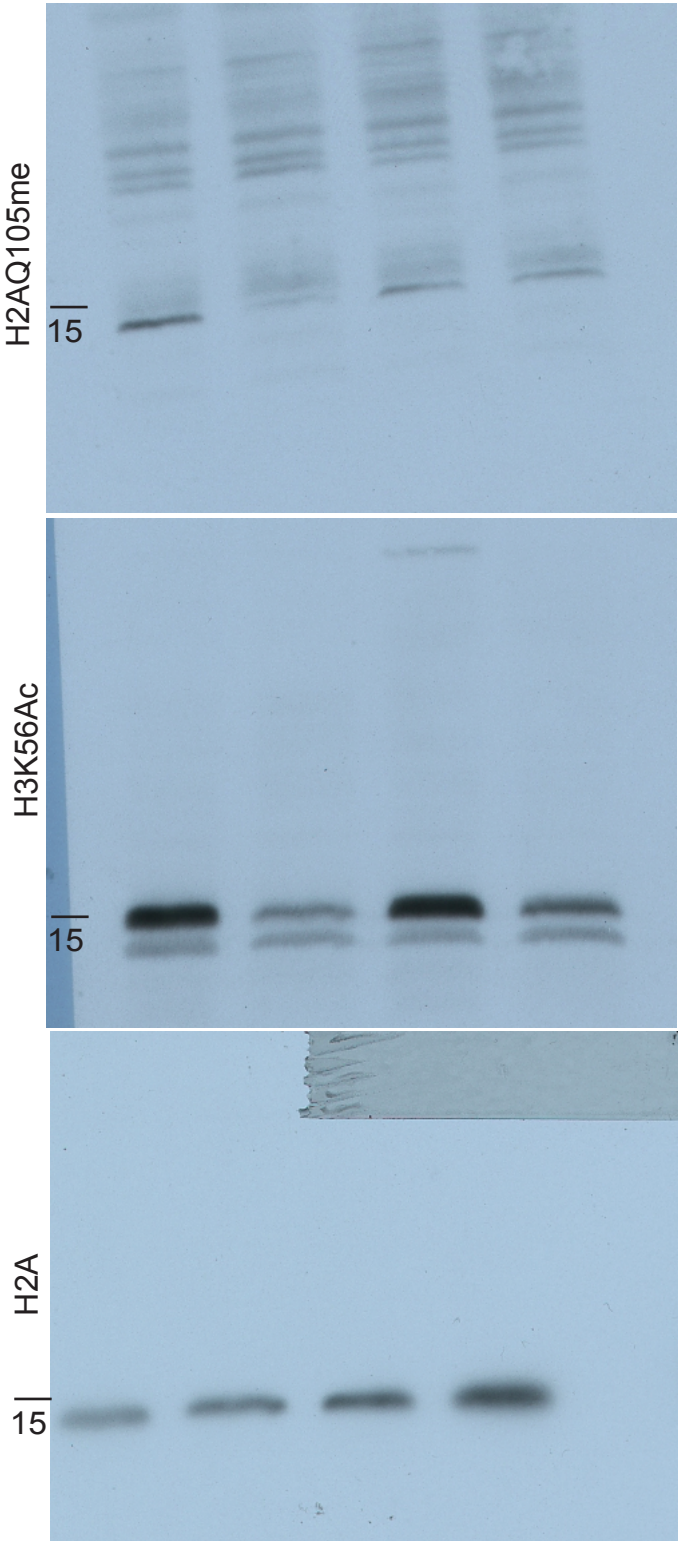

Figure 1C

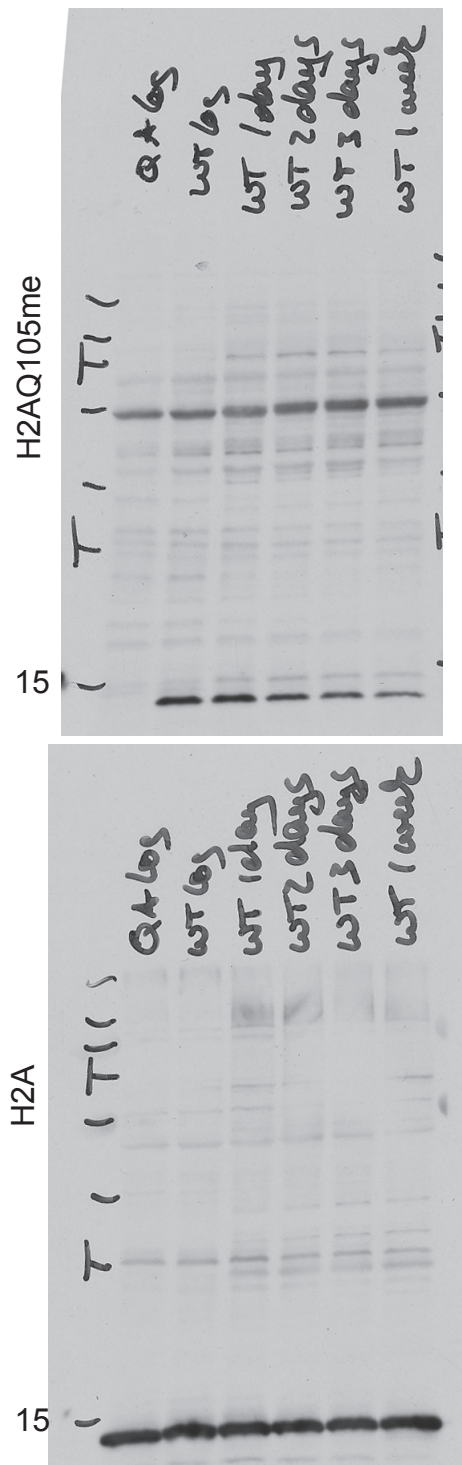

Figure 1D

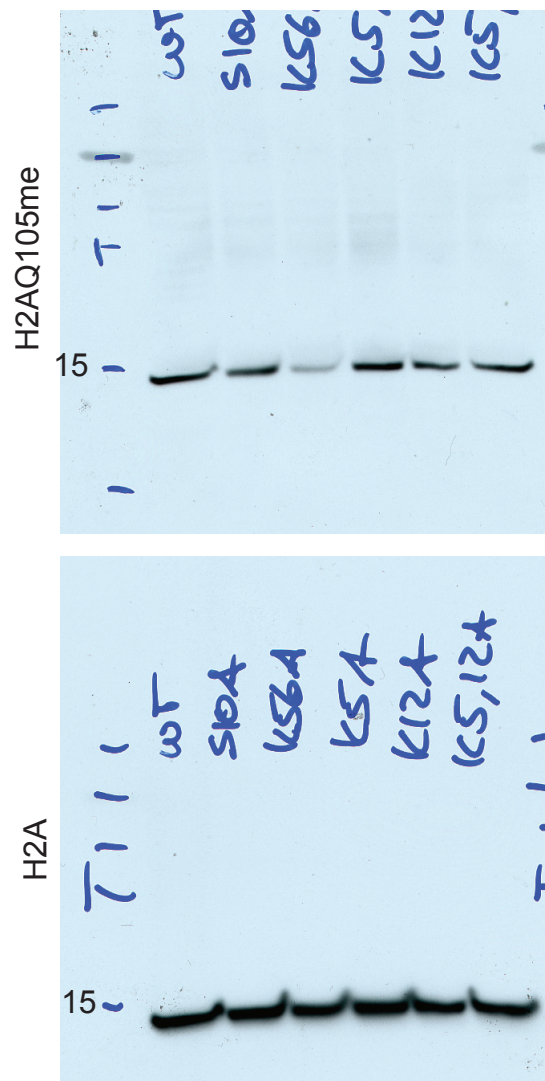

Figure 1E

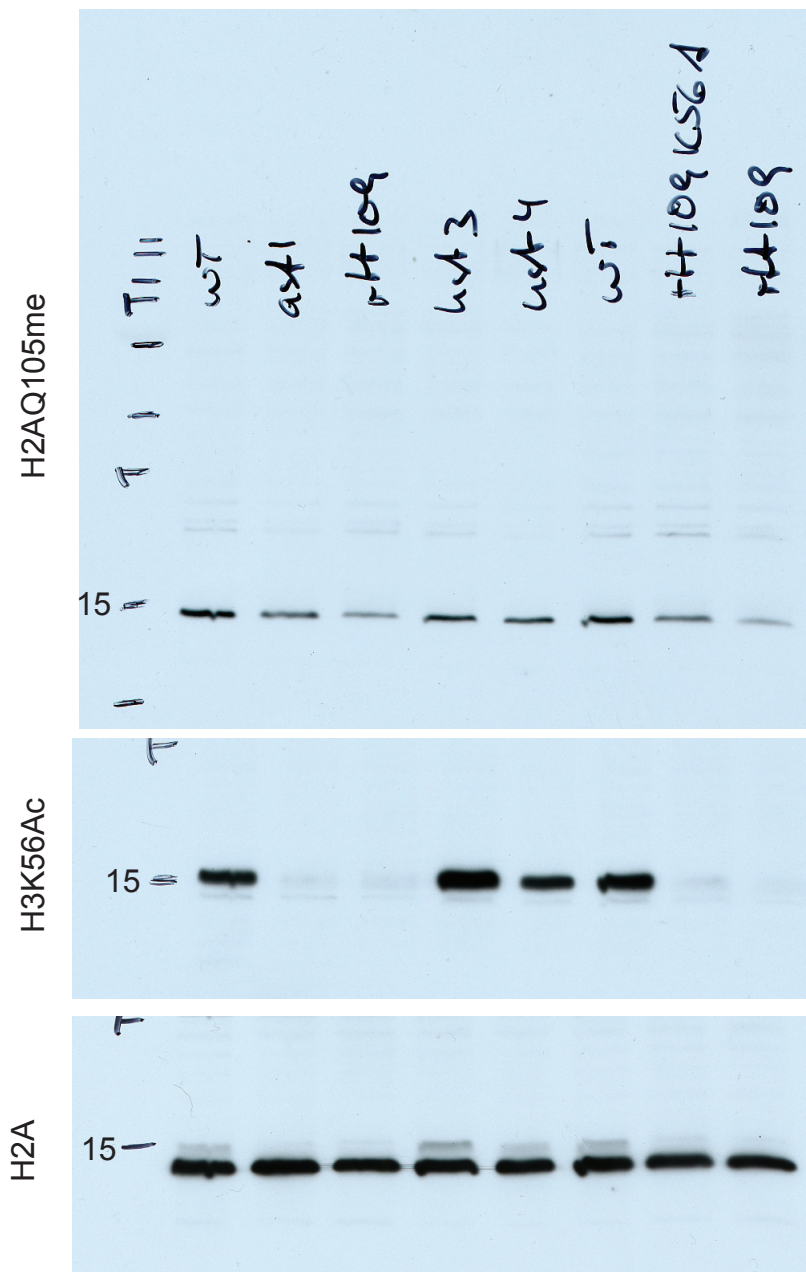

Supplement: Supplementary file 6 — Source Data for Figure 1 [file EMBR-22-e52435-s007.pdf]

Figure 3B

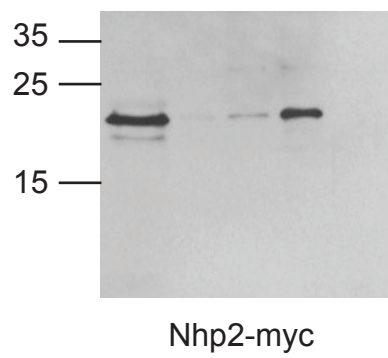

Figure 3D

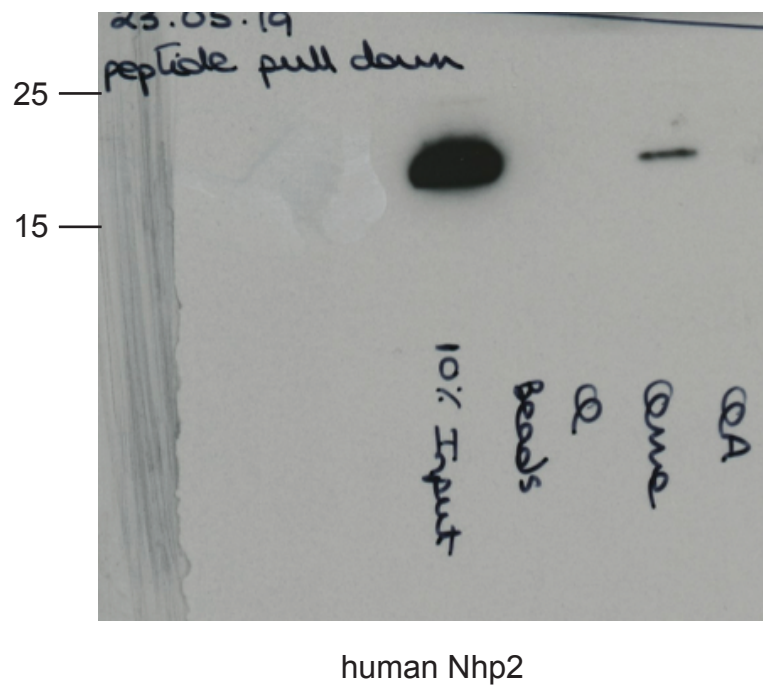

Supplement: Supplementary file 7 — Source Data for Figure 3 [file EMBR-22-e52435-s008.pdf]
