# Supplementary material for: Nhp2 is a reader of H2AQ105me and part of a network integrating metabolism with rRNA synthesis
Source: EMBO Rep. 2021 Aug 19;22(10):e52435. doi: 10.15252/embr.202152435 (PMC8490984; doi:10.15252/embr.202152435)
Supplement: Supplementary file 8 — Source Data for Figure 4 [file EMBR-22-e52435-s004.pdf]

Figure 4A

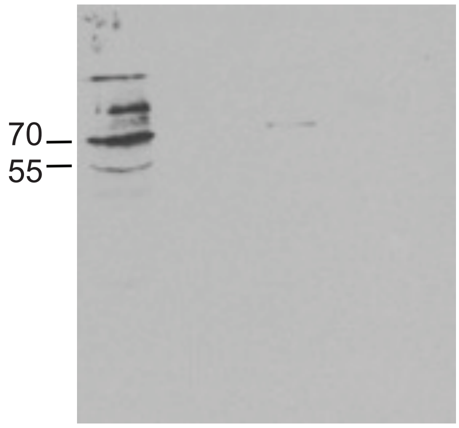

Cbf5-TAP

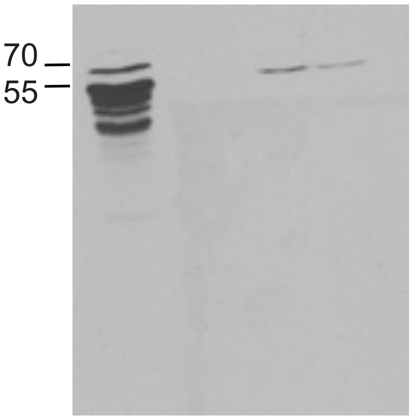

Gar1-TAP

Figure 4G

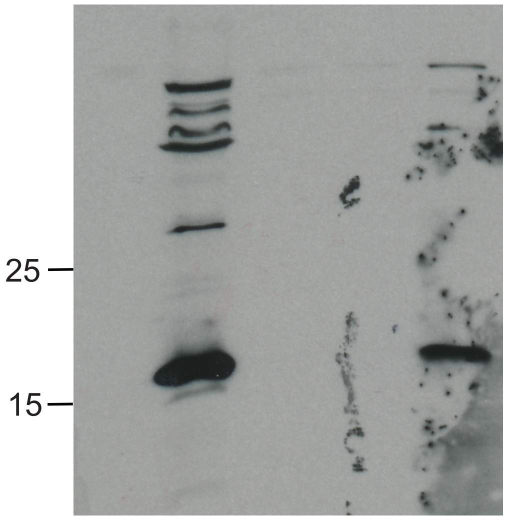

Nhp2

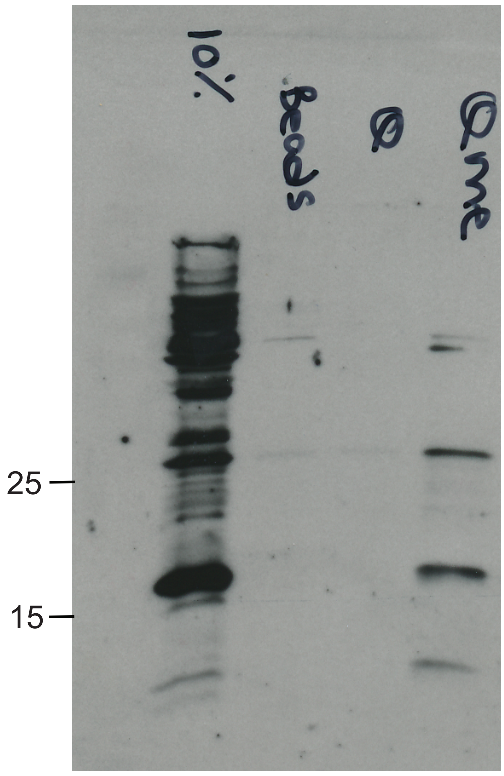

Utp11 - Nhp2 signal remained due to high exposure for Utp11 and insufficient stripping of NHP2

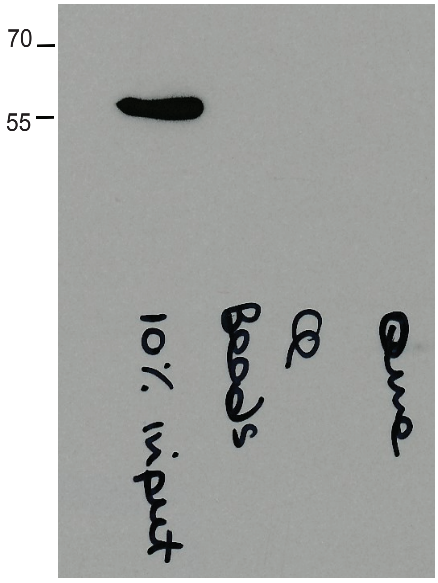

Dyskerin

Representative Northern Blots for the analyses shown in Figures 4H and 4I.

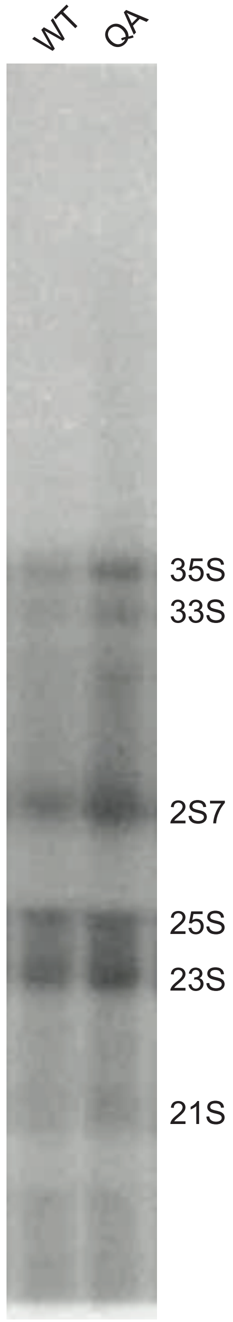

21S probe

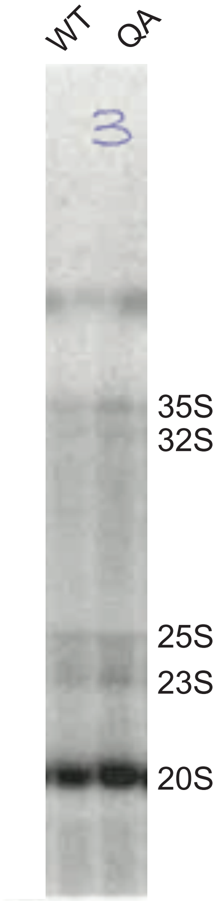

20S probe

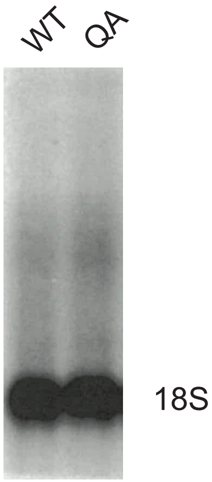

18S probe

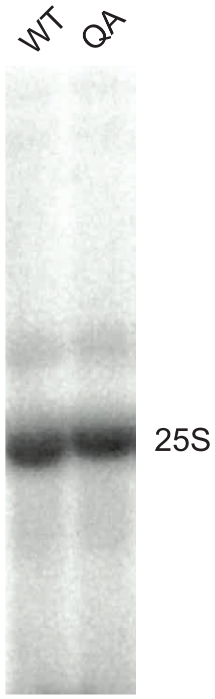

25S probe

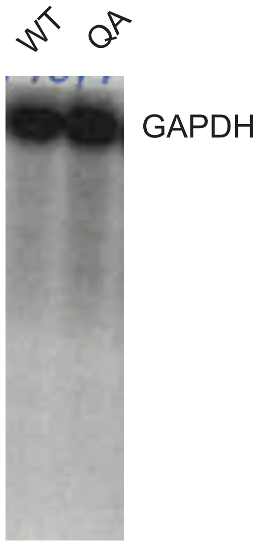

GAPDH probe
