# Supplementary material for: Nhp2 is a reader of H2AQ105me and part of a network integrating metabolism with rRNA synthesis
Source: EMBO Rep. 2021 Aug 19;22(10):e52435. doi: 10.15252/embr.202152435 (PMC8490984; doi:10.15252/embr.202152435)
Supplement: Supplementary file 9 [file EMBR-22-e52435-s002.pdf]

EMBO reports

Nhp2 is a reader of H2AQ105me and part of a network integrating metabolism with rRNA synthesis

Julia Mawer, Jennifer Maassen, Christina Reichert, Niklas Grabenhorst, Constantine Mylonas, and Peter Tessarz
DOI: 10.15252/embr.202152435

Corresponding author(s): Peter Tessarz (ptessarz@age.mpg.de)

Review Timeline:

Submission Date:	11th Jan 21
Editorial Decision:	10th Feb 21
Revision Received:	7th Jun 21
Editorial Decision:	20th Jul 21
Revision Received:	2nd Aug 21
Accepted:	6th Aug 21

Editor: Achim Breiling

Transaction Report:

(Note: With the exception of the correction of typographical or spelling errors that could be a source of ambiguity, letters and reports are not edited. Depending on transfer agreements, referee reports obtained elsewhere may or may not be included in this compilation. Referee reports are anonymous unless the Referee chooses to sign their reports.)

1st Editorial Decision 10th Feb 2021

Dear Dr. Tessarz,

Thank you for the submission of your research manuscript to EMBO reports. We have now received the reports from the three referees that were asked to evaluate your study, which can be found at the end of this email.

As you will see, the referees think that these findings are of interest. However, they have several comments, concerns and suggestions, indicating that a major revision of the manuscript is necessary to allow publication of the study in EMBO reports. As the reports are below, and all their points need to be addressed, I will not detail them here.

Given the constructive referee comments, we would like to invite you to revise your manuscript with the understanding that all referee concerns must be addressed in the revised manuscript or in the detailed point-by-point response. Acceptance of your manuscript will depend on a positive outcome of a second round of review. It is EMBO reports policy to allow a single round of revision only and acceptance of the manuscript will therefore depend on the completeness of your responses included in the next, final version of the manuscript.

Revised manuscripts should be submitted within three months of a request for revision. We are aware that many laboratories cannot function at full efficiency during the current COVID-19/SARS-CoV-2 pandemic and we have therefore extended our 'scooping protection policy' to cover the period required for full revision. Please contact me to discuss the revision should you need additional time, and also if you see a paper with related content published elsewhere.

When submitting your revised manuscript, please also carefully review the instructions that follow below.

PLEASE NOTE THAT upon resubmission revised manuscripts are subjected to an initial quality control prior to exposition to re-review. Upon failure in the initial quality control, the manuscripts are sent back to the authors, which may lead to delays. Frequent reasons for such a failure are the lack of the data availability section (please see below) and the presence of statistics based on n=2 (the authors are then asked to present scatter plots or provide more data points).

When submitting your revised manuscript, we will require:

1) a .docx formatted version of the final manuscript text (including legends for main figures, EV figures and tables), but without the figures included. Please make sure that changes are highlighted to be clearly visible. Figure legends should be compiled at the end of the manuscript text.

2) individual production quality figure files as .eps, .tif, .jpg (one file per figure), of main figures and EV figures. Please upload these as separate, individual files upon re-submission.

The Expanded View format, which will be displayed in the main HTML of the paper in a collapsible format, has replaced the Supplementary information. You can submit up to 5 images as Expanded View. Please follow the nomenclature Figure EV1, Figure EV2 etc. The figure legend for these should be included in the main manuscript document file in a section called Expanded View Figure Legends after the main Figure Legends section. Additional Supplementary material should be supplied as a single pdf file labeled Appendix. The Appendix should have page numbers and needs

to include a table of content on the first page (with page numbers) and legends for all content. Please follow the nomenclature Appendix Figure Sx, Appendix Table Sx etc. throughout the text, and also label the figures and tables according to this nomenclature.

For more details, please refer to our guide to authors:
http://www.embopress.org/page/journal/14693178/authorguide#manuscriptpreparation

See also our guide for figure preparation:
http://wol-prod-cdn.literatumonline.com/pb-assets/embo-site/EMBOPress_Figure_Guidelines_061115-1561436025777.pdf

3) a .docx formatted letter INCLUDING the reviewers' reports and your detailed point-by-point responses to their comments. As part of the EMBO Press transparent editorial process, the point-by-point response is part of the Review Process File (RPF), which will be published alongside your paper.

4) a complete author checklist, which you can download from our author guidelines (https://www.embopress.org/page/journal/14693178/authorguide). Please insert page numbers in the checklist to indicate where the requested information can be found in the manuscript. The completed author checklist will also be part of the RPF.

Please also follow our guidelines for the use of living organisms, and the respective reporting guidelines: http://www.embopress.org/page/journal/14693178/authorguide#livingorganisms

5) that primary datasets produced in this study (e.g. RNA-seq, ChIP-seq, structural and array data) are deposited in an appropriate public database. If no primary datasets have been deposited, please also state this in the respective section (e.g. 'No primary datasets have been generated and deposited'), see below.

See also: http://embor.embopress.org/authorguide#datadeposition

Please remember to provide a reviewer password if the datasets are not yet public.

The accession numbers and database should be listed in a formal "Data Availability " section (placed after Materials & Methods) that follows the model below. This is now mandatory (like the COI statement). Please note that the Data Availability Section is restricted to new primary data that are part of this study.

Data availability

The datasets produced in this study are available in the following databases:

- RNA-Seq data: Gene Expression Omnibus GSE46843 (https://www.ncbi.nlm.nih.gov/geo/query/acc.cgi?acc=GSE46843)
- [data type]: [name of the resource] [accession number/identifier/doi] ([URL or identifiers.org/DATABASE:ACCESSION])

*** Note - All links should resolve to a page where the data can be accessed. ***

Moreover, I have these editorial requests:

6) We strongly encourage the publication of original source data with the aim of making primary data more accessible and transparent to the reader. The source data will be published in a separate source data file online along with the accepted manuscript and will be linked to the relevant figure. If you would like to use this opportunity, please submit the source data (for example scans of entire gels or blots, data points of graphs in an excel sheet, additional images, etc.) of your key experiments together with the revised manuscript. If you want to provide source data, please include size markers for scans of entire gels, label the scans with figure and panel number, and send one PDF file per figure.

7) Our journal encourages inclusion of *data citations in the reference list* to directly cite datasets that were re-used and obtained from public databases. Data citations in the article text are distinct from normal bibliographical citations and should directly link to the database records from which the data can be accessed. In the main text, data citations are formatted as follows: "Data ref: Smith et al, 2001" or "Data ref: NCBI Sequence Read Archive PRJNA342805, 2017". In the Reference list, data citations must be labeled with "[DATASET]". A data reference must provide the database name, accession number/identifiers and a resolvable link to the landing page from which the data can be accessed at the end of the reference. Further instructions are available at:
http://www.embopress.org/page/journal/14693178/authorguide#referencesformat

8) Regarding data quantification and statistics, can you please specify, where applicable, the number "n" for how many independent experiments (biological replicates) were performed, the bars and error bars (e.g. SEM, SD) and the test used to calculate p-values in the respective figure legends. Please provide statistical testing where applicable, and also add a paragraph detailing this to the methods section. See:
http://www.embopress.org/page/journal/14693178/authorguide#statisticalanalysis

9) Please also note our new reference format:
http://www.embopress.org/page/journal/14693178/authorguide#referencesformat

10) Please add a conflict-of-interest statement and a paragraph detailing the author contributions to the manuscript (before the acknowledgements).

11) Please also add up to five key words to the title page.

I look forward to seeing a revised version of your manuscript when it is ready. Please let me know if you have questions or comments regarding the revision.

Yours sincerely,

Achim Breiling
Editor
EMBO Reports

Referee #1:

Firstly, the authors confirm that H2AQ105 methylation is cell cycle-dependent and requires proliferation. They then identify preliminary genetic interactions between H2AQ105 methylation and ribosomal genes, and mTor pathway genes (see comment #2). The mTor inhibitor, rapamycin,

leads to a loss in proliferation, pol I occupancy, and H2AQ105me. Similarly, inhibiting H3K56ac led to a reduction in H2AQ105me. However, as the authors also note, the loss of H2AQ105me in both cases may be a downstream consequence of diminished rRNA transcription rather than a direct effect. A quantitative mass spec experiment reveals a list of 139 candidate proteins that preferentially bind H2AQ105me, Nhp2 as a direct reader of Q105me in yeast. This dataset is a valuable resource that may be used to identify other readers of H2AQ 105me and proteins that preferentially bind the unmodified H2AQ105. Other peptide pull-down experiments provide more in vitro evidence suggesting that Nhp2 is likely a direct reader of Q105me in yeast and possibly in humans (see comment #6). Finally, the authors show no detectable link between H2AQ105me and pseudouridylation of rRNA, which is the main function of Nhp2 (see comment #4). The authors hypothesized that H2AQ105 methylation might be necessary to process the small ribosomal subunit (SSU) because SSU processome components interact with H2AQ105me Nhp2 (see comment #7). However, the biological significance of Nhp2 recruitment to H2AQ105me remains unanswered.

Overall, a better understanding of the readers of the rDNA-specific H2AQ105me histone modification will lead to an improved understanding of the many layers of ribosomal RNA regulation and possibly inform similar processes elsewhere in the genome.
Major comments:

1) It is not clear if Nhp2 recruitment to H2AQ105me has implications for rRNA synthesis, as the title implies.

2) Line 118: A summary of what is known about the H2AQ105A mutant strain from Tessarz et al., 2014 and other published work would greatly facilitate interpreting the SGA results. Does this mutant phenocopy the unmodified Q105? In Tessarz et al., 2014 this mutation appears to phenocopy Q105me with regards to FACT binding.
Also, the SGA results are unclear. How can one explain the apparent partial rescue of cell growth in ribosomal and mTor KO cells? Is it surprising that one did not observe a negative genetic interaction?
Ideally, one should confirm SGA results with small-scale spot tests for ribosomal and mTor genes to validate the relevant hits from the large-scale SGA.

3) Mutating H3K56 to alanine leads to reduced Q105me, but the authors speculate that the dependency of Q105 methylation on H3K56ac is due to diminished rRNA transcription (lines 158-159). The authors tested Nop1 occupancy to address this indirectly. One might directly test this hypothesis with assays similar to the ones shown in Supplemental Figure 1 (growth curve and Pol I ChIP) or a nuclear run-on experiment showing decreased nascent rRNA transcription.
Line 155: hst3/4 deletion strains have higher levels of H3K56ac, but H2AQ105me levels are the same as WT. Is it surprising that H2AQ105me levels are not rising with increasing H3K56ac?

4) Based on the ChIP-qPCR, H2AQ105A mutants still demonstrate 50% Nhp2 recruitment compared to WT H2AQ105. However, in vitro experiments show no binding of recombinant Nhp2 or Nhp2-myc to the Q105A mutant peptide. This raises the question of whether Nhp2 has different modes of recruitment to the rDNA, besides interacting with H2AQ105me.

5) Based on the lane labels in Figure 4D, it appears that this assay evaluates the binding of human Nhp2 with the yeast peptide containing Q105me, rather than the human Q104me peptide. Please clarify which peptide was used in this panel. If the yeast peptide was indeed used, it is not surprising that the human Nhp2 binds weaker to the yeast Q105me peptide compared to yeast Nhp2. Unless

yeast and human Nhp2 are identical at the amino acid level, caution is warranted when interpreting these results.

6) The paper's main novelty is the identification of Nhp2 as a direct reader of H2AQ105me. However, all the data supporting this point are based on similar pull-down experiments that are coupled to mass spectrometry or PAGE/WB. Ideally, the direct interaction between Q105me and Nhp2 should be verified using a different ITC or equivalent assay.

7) The authors found 17 candidate proteins (enriched in SSU) interacting with each of Nhp2 and H2AQ105me. This raises a question; Do H2AQ105A mutants have defects in the SSU processing? A Northern would show any rRNA processing defects that might arise if SSU processing enzymes (such as Mpp10, Imp4, Utp11 (line 290)) are not recruited efficiently to the pre-rRNA. Similarly, RNA IP may show if these factors' recruitment to the pre-rRNA is affected in the H2AQ105A mutants.

8) Recommendation. One wonders whether differential gene expression analysis with RNA-seq from WT and H2AQ105A strains would reveal compensatory transcriptomic changes and provide insight into this network.

Minor comments:

-Some abbreviations are not defined throughout the text. For example, see Line 89 Qme; Figure 1B Noc, and Figure 2A NTS2, 5' ETS, ITS1. In general, the figures are poorly described in the legends. It would greatly help the audience if the authors included more details in the figure legends. For example, it's not clear what red versus grey represents in Figure 1E. What are the error bars in the ChIP-qPCR graphs (like in Figure S2)?

-For clarity, it would be better to abbreviate RNA Pol I with space rather than "RNA PolI," which is frequently referred to throughout the manuscript, as this might be confused with RNA pol II.

-Line 103-107: Please explain in the methods section how the samples were collected after being grown deep into stationary phase but then "sampled during log-phase of growth."

-Line 130: "addition of rapamycin led to a loss of RNA polI from the rDNA locus" is shown in Figure S1B and not in S1A. "... and a stop in proliferation" is shown in figure S1A and not in S1B.

-Figure 3C and line 195: Are the ribosome biogenesis factors enriched in certain stages of ribosome biogenesis, i.e., early vs. late? Can the authors classify them according to the complexes they belong to, such as t-UTP, UTP-B, MPP10 complex, etc?

-Figure 3C is not very meaningful without labels for individual proteins or networks, such as those noted in the legend.

-Line 230: "mammalian cell lysate." What cell type was used in these experiments? This information should be mentioned in the results as well as the methods section.

-How many biological replicates were included in the mass spectrometry experiments? What about technical replicates? The methods section seems to be missing these.

-What platform were the ChIP samples sequenced on (i.e., Illumina HiSeq, MiSeq, etc.)? What is the read length? Was input also sequenced? Please show the track for input in Figure 4E. The y-axis

label for the ChIP-seq tracks is missing in Figure 4E. I'm wondering if the two depicted 35S rDNA copies are identical? Nhp2 coverage seems identical at the two 35S regions, whereas the H2AQ105me track looks very much different? Are the Nhp2 and H2AQ105me tracks mapped the same way, using the same parameters?

-Line 212: Shouldn't it refer to Figure 4B?

Referee #2:

In this report, the authors provide evidence that the poorly understood histone modification H2AQ105me, which localizes to solely to the rDNA, is bound by the protein Nhp2 that functions in ribosomal biogenesis. They further demonstrate that H2AQ105me levels are cell-cycle dependent, and that they specifically depend on cell proliferation as arresting cells at any stage of the cell cycle reduces the global abundance of this histone modification. They further provide evidence that H2AQ105me depends on both Pol I transcription, as well as the TORC1 regulated Rtt109-Asf1 pathway, which previously was shown to regulate H3K56ac at the rDNA and promote Pol I transcription.

Overall, the work presented in this manuscript is of high quality, and the results presented would be of significant interest to the readership of EMBO Reports. However, there are a few concerns that need to be addressed before acceptance of the manuscript. These are described below.

1) The authors discuss the requirement for cell proliferation in the maintenance of H2AQ105me, and they provide data that arresting cells in G1, early S-phase, and at the G2/M boundary reduces its abundance. While they specifically point out that a previous study demonstrated that S-phase arrest impairs Pol I transcription, they do not provide an explanation for why this modification (which seems to depend on Pol I transcriptional activity) also is reduced in G1 and/or G2/M arrested cells. Have they (or anyone else) examined the transcriptional activity of Pol I in G1 or G2/M arrested cells? This point is especially important since most anabolism occurs in G1. The authors should address and/or elaborate on this issue as it might be expected that cells in G1 would still have transcriptionally active Pol I, and hence H2AQ105me.

2) The results for Nhp2 as a binder of H2AQ105me are strong, but I do not believe they are 100% convincing. It would be really helpful if the authors actually identified the region of Nhp2 that is responsible (using their recombinant protein approach and in vitro binding as one possibility), made mutations in this region, and then expressed the mutant in vivo to test if it fails to bind to the rDNA and/or fails to pull-down H2AQ105me histone. Combined with the data they already have presented, this would unequivocally demonstrate that Nhp2 is an in vivo binder for this epigenetic modification.

Technical concern:
1) Peptide pull-down experiments can be difficult to perform since normalizing the amount of each distinct peptide used for the pull-downs can be somewhat variable. This issue becomes especially important for the experiments in this report since these peptides are histone peptides, which typically are highly charged and tend to stick to proteins non-specifically. Unequal amounts of the different peptides could potentially skew the results. The authors should demonstrate for their peptide pull-down experiments equal amounts of the peptides present, at least in an "input" type format. Since the peptides are biotin-labeled, a simple dot-blot combined with streptavidin-HRP

immunoblot would suffice, which would demonstrate to the reviewer and/or reader that equal amounts of the different peptides are being tested.

Referee #3:

The manuscript "Nhp2 is a reader of H2AQ105me and part of a network integrating metabolism with rRNA synthesis" proposes that Nhp2 specifically recognizes H2AQ105me at rDNA as part of the rRNA synthesis pathway. The evidence that demonstrates Nhp2 and H2AQ105me interact is found in figure 4, where a H2AQ105A mutant has reduced Nhp2 binding relative to wild-type by ChIP-qPCR. Nhp2 was further shown to specifically interact with methylated H2AQ105 proteins through peptide pulldowns. Interestingly, the authors determined that this chromatin-protein interaction was not tied to the known function of Nhp2 in pseudouridylation of rRNA, but instead propose a new role for Nhp2 in small ribosomal subunit (SSU) processing. The evidence provided in figure 5 supports the hypothesis that H2AQ105me is not essential for pseudouridylation; however, support for Nhp2's role in SSU processing is limited to analyzing mass spectrometry analyses of Nhp2 and H2AQ105me. The findings from this study would be strengthened by directly testing the hypothesis that Nhp2 reads H2AQ105me as part of the SSU processing pathway. The first 2 figures of the study focus on H2AQ105me regulation instead of the Nhp2-H2AQ105me interaction. The authors propose that H2AQ105me is cell-cycle regulated in a similar manner to H3K56 acetylation, and that H3K56 acetylation and the Tor signaling pathway are key regulators of H2AQ105me at rDNA. This data, as presented, is not strongly related to the Nhp2 finding and detracts from the overall story; however, in the discussion the authors draw an interesting parallel between H3K56 acetylation and SSU processing that, if explored, could greatly enhance their findings and focus their study. Overall, the main finding that Nhp2 reads H2AQ105me is supported by the data, but the purpose of this interaction is not adequately explored and thus it might be a bit premature to call it a bonafide reader. Additionally, the initial data characterizing H2AQ105me regulation could be more strongly tied to the main finding by exploring the relationship between H3K56 acetylation, H2AQ105me regulation, and Nhp2 function in SSU processing.

Major concerns:
1. The SGA analysis provided in figure 1 is poorly represented by the images shown. In the image meant to demonstrate rescue with H2AQ105A, all colonies throughout the image appear larger. Generating growth curves comparing H2AQ105A rescued candidates to WT-H2AQ105me candidates would provide more definitive evidence, however this data would not strengthen the Nhp2 hypothesis and could be removed.
2. As presented, the first two figures detract from the focus of the manuscript, which is the Nhp2-H2AQ105me interaction. Rather than focusing on cell-cycle regulation in figure 1, the authors could focus on the shared cell-cycling between H2AQ105me and H3K56 (which is nicely shown in figure 1 panel A) before moving into regulation of H2AQ105me by H3K56 acetylation.
3. It is unclear how the Nop1 ChIP-qPCR data presented in figure 3 is related to the Nhp2-H2AQ105me story. This data could be removed or should be further explained.
4. In figure 4, the authors provide ChIP-seq data for Nhp2 and H2AQ105me. To better interpret this data, the authors should provide a scale for the Y-axis of the ChIP-seq peaks. A more detailed explanation of the experimental approach, including the number of samples sequenced and how the mapping of reads to the repetitive rDNA locus was conducted, would be beneficial. Additionally, this data prompts the question of Nhp2 binding throughout the genome. Does Nhp2 bind only to the rDNA repeats, or does it bind to other regions? Could the authors explain why there are no Nhp2 peaks at the smaller H2AQ105me peaks shown along chromosome 12?

5. In figure 5, the authors demonstrate that Nhp2 does not interact with H2AQ105me as part of the pseudouridylation processing of rRNA. From their mass spectrometry data, they propose that Nhp2 is reading this chromatin mark to function in SSU processing. They also mention that H3K56 acetylation regulation, which they demonstrate regulates H2AQ105me in figure 2, is involved in SSU processing. If H3K56 recruitment is essential for SSU recruitment at rDNA, then the authors may have identified Nhp2's reading H3K56-regulated H2AQ105me as a key component of this pathway. The authors should examine small ribosomal subunit processing in the H2AQ105A mutant they have generated and in a Nhp2 mutant. This could be done by looking at SSU factor recruitment, such as Utp9, or by analyzing 40S levels in these two mutants.

Minor concerns:
1. The qPCR data in figure 4A appears to be labeled incorrectly. The H2AQ105A bars are black but the key indicates they should be orange. This error (at least I think its an error) makes the data appear opposite of their written conclusion.
2. In the introduction (lines 36-40), the authors imply that chromatin modifications at rDNA regulate RNA pol I. While this may be true, the example they provide (rapamycin treatment reduces both RNA pol I transcription and acetylation levels) does not support this statement. The example provided regarding H3K56 acetylation from the Chen et al 2012 study (line 156-157) better supports this statement. While the levels of RNAP1 by ChIP are clearly reduced, there is no direct measurement of transcription to support the claim.
3. In panel E of figure 2, the 28S line appears dashed. It is not clear if this line is dashed for a particular reason or if it should solid like the other lines.
4. The authors identify five potential readers from their MS data described in figure 3. A more detailed explanation of what characteristics they used to identify potential readers would be helpful.
5. Line 256 should have "extent" instead of "extend."
6. Line 300 should say "maintaining" instead of "maintain."
7. Line 305-306 is grammatically incorrect and should be rephrased.

Evaluation:
1. Does this manuscript report a single key finding? YES/NO
YES, this report identifies Nhp2 as a reader of H2AQ105me for a new role in rRNA biogenesis that has not previously been attributed to Nhp2.
2. Is the reported work of significance (YES), or does it describe a confirmatory finding or one that has already been documented using other methods or in other organisms etc (NO)?
a. YES, the work is significant in that it reports a new function for the essential and conserved rRNA processing protein Nhp2.
3. Is it of general interest to the molecular biology community? YES
4. If YES, please say why, in a single sentence. If NO, please state which more specialized community you feel it is aimed at (or none), in a single word or phrase.
a. The work identifies a new function for rRNA processing protein Nhp2, highlighting a new role for a conserved protein in an essential pathway.
4. Is the single major finding robustly documented using independent lines of experimental evidence (YES), or is it really just a preliminary report requiring significant further data to become convincing, and thus more suited to a longer¬format article (NO)? YES/NO
a. Yes, the finding that Nhp2 interacts with H2AQ105me outside of its established function in rRNA processing is robustly documented.
Detailed report
1. What are the major claims and how significant are they?
a. The major finding is that Nhp2 recognizes H2AQ105me at rDNA for a role in rRNA processing

outside of Nhp2's currently accepted role in pseudouridylation. This is a significant new finding for Nhp2's function in ribosome biogenesis.

b. The authors provide evidence that H2AQ105me is regulated by Tor signaling and H3K56 acetylation. The evidence provided is sufficient for this claim, however the claim itself detracts from the focus on Nhp2. The authors previously reported that H2AQ105me requires RNA pol I transcription for deposition. RNA pol I is known to be regulated by Tor signaling, which makes the regulation of H2AQ105me by Tor an expected finding. The data suggesting H3K56 acetylation regulates H2AQ105me is interesting but is not explored enough to make significant conclusions.

2. Are the claims novel and convincing?

The claim that Nhp2 recognizes H2AQ105me is novel and is supported by multiple lines of evidence presented in figures 3 and 4. The additional claim that Nhp2 does not recognize this histone modification as part of its established role in pseudouridylation is novel and supported by evidence in figure 5. The suggestion that Nhp2 may function in SSU processing is interesting but limited to assumptions made from quantitative mass spectrometry.

3. Are the claims appropriately discussed in the context of earlier literature?

The literature provided in the introduction and discussion provides appropriate context for the study.

4. Who will be interested and why?

This paper will interest cell biologists interested in understanding ribosome biogenesis, particularly at the level of rRNA processing. Additionally, this paper will strongly interest molecular biologists with an interest in epigenetics, particularly those studying the regulatory impact of epigenetic modifications at the rDNA on rDNA's function in ribosome biogenesis.

5. Does the paper stand out in some way from the others in its field?

The paper provides new insight into Nhp2's function in the rRNA production pathway by demonstrating its interaction with H2AQ105me using standard molecular biology techniques and is guided by established literature regarding the ribosome biogenesis pathway. The paper does not stand out inappropriately from its field.

6. Are the experimental data of sufficient quality to justify the conclusions?

The experimental data supporting the function of Nhp2 as a reader of H2AQ105me is sufficiently provided in figure 4 by presenting decreased Nhp2 binding in an H2AQ105A mutant and peptide pulldowns specifically showing Nhp2 and H2AQ105me interactions. The addition of H3K56, which appears tied to H2AQ105me, nicely supports the specificity of the Nhp2-H2AQ105me interaction. The hypothesis that Nhp2 is involved in SSU is weak and merely suggested by the mass spectrometry data in figures 3 and 5. While the authors clearly note this, the overall study could be strengthened by further addressing the role of the Nhp2-H2AQ105me interaction in SSU processing. The SGA data provided in figure 1 is not sufficient quality and is not essential to the main findings of the study.

We would like to thank the reviewers for their overall appreciation of our study and the very clear and constructive feedback on our manuscript. We have now addressed all concerns and highlight our approach in the point-by-point answer to the individual points below. Reviewer's comments are marked in *italics*.

Referee #1:
Firstly, the authors confirm that H2AQ105 methylation is cell cycle-dependent and requires proliferation. They then identify preliminary genetic interactions between H2AQ105 methylation and ribosomal genes, and mTor pathway genes (see comment #2). The mTor inhibitor, rapamycin, leads to a loss in proliferation, pol I occupancy, and H2AQ105me. Similarly, inhibiting H3K56ac led to a reduction in H2AQ105me. However, as the authors also note, the loss of H2AQ105me in both cases may be a downstream consequence of diminished rRNA transcription rather than a direct effect. A quantitative mass spec experiment reveals a list of 139 candidate proteins that preferentially bind H2AQ105me, Nhp2 as a direct reader of Q105me in yeast. This dataset is a valuable resource that may be used to identify other readers of H2AQ 105me and proteins that preferentially bind the unmodified H2AQ105. Other peptide pull-down experiments provide more in vitro evidence suggesting that Nhp2 is likely a direct reader of Q105me in yeast and possibly in humans (see comment #6). Finally, the authors show no detectable link between H2AQ105me and pseudouridylation of rRNA, which is the main function of Nhp2 (see comment #4). The authors hypothesized that H2AQ105 methylation might be necessary to process the small ribosomal subunit (SSU) because SSU processome components interact with H2AQ105me Nhp2 (see comment #7). However, the biological significance of Nhp2 recruitment to H2AQ105me remains unanswered.
Overall, a better understanding of the readers of the rDNA-specific H2AQ105me histone modification will lead to an improved understanding of the many layers of ribosomal RNA regulation and possibly inform similar processes elsewhere in the genome.

We would like to thank the reviewer for this positive assessment of our manuscript. We have answered the individual concerns below.

Major comments:

1) It is not clear if Nhp2 recruitment to H2AQ105me has implications for rRNA synthesis, as the title implies.

<u>Response:</u> We agree with the reviewer that we did not provide insights into a potential role for H2AQ105me in rRNA processing and ribosome biogenesis in general. We focused our revision exactly on this point as we also feel that our original manuscript fell short in this regard. To this end, we performed an RNA-seq experiment comparing WT and H2AQ105A strains under logarithmic growth conditions. While the overall changes in the transcriptome were very subtle, gene set enrichment analysis did highlight an enrichment for terms involved in ribosome biogenesis and, in particular, the SSU processome (new Figure 3E). We also attempted to address the involvement of the H2AQ105me-Nhp2 axis more directly and performed Northern Blots to quantify processing intermediates of rRNA in wild type and H2AQ105A mutants. While we did not observe any changes in the steady-state levels of rRNA between the 2 strains (new Figure EV 4), we specifically observed a small but

significant enrichment of processing intermediates of the 18S rRNA in H2AQ105A mutants (new Figures 5H,I), providing evidence of H2AQ105me's involvement in rRNA processing. We added a paragraph each in the results/discussion section, in which we discuss this finding and the likely involvement of Nhp2 (lines 281-288 and 323-333).

2) Line 118: A summary of what is known about the H2AQ105A mutant strain from Tessarz et al., 2014 and other published work would greatly facilitate interpreting the SGA results. Does this mutant phenocopy the unmodified Q105? In Tessarz et al., 2014 this mutation appears to phenocopy Q105me with regards to FACT binding.

Response: We agree and have added a section in this paragraph summarising the relevant and known information about the H2AQ105A mutant.

Also, the SGA results are unclear. How can one explain the apparent partial rescue of cell growth in ribosomal and mTor KO cells? Is it surprising that one did not observe a negative genetic interaction? Ideally, one should confirm SGA results with small-scale spot tests for ribosomal and mTor genes to validate the relevant hits from the large-scale SGA.

Response: We fully agree that these results were really surprising and we believe are worth publishing. However, we now performed a small-scale screen of some candidates. We had problems generating some strains in our W303 background, but of the three we managed to generate, only one (rpl12) recapitulated the results (new Figure 1G). We also tried to gain more insight into the role of mTor signalling in the genetic interaction with H2A105A and performed spot tests using various concentration ranges of rapamycin. In this context, we did not observe any difference in growth between a WT and an H2AQ105A mutant (R-Figure 1). As also other reviewers and the overall evaluation points to the SGA as a weakness of the manuscript without much of an addition to the findings, we would offer to remove these data from the manuscript if necessary.

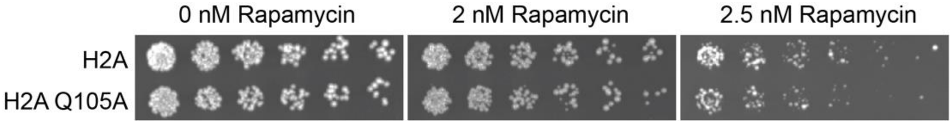

R-Figure 1: Spot test of S. cerevisiae expressing either H2A or H2AQ105A as sole source of H2A. Cells were diluted 5-fold and equal cell numbers were spotted on YPD supplemented with the indicated rapamycin concentrations.

3) Mutating H3K56 to alanine leads to reduced Q105me, but the authors speculate that the dependency of Q105 methylation on H3K56ac is due to diminished rRNA transcription (lines 158-159). The authors tested Nop1 occupancy to address this indirectly. One might directly test this hypothesis with assays similar to the ones shown in Supplemental Figure 1 (growth curve and Pol I ChIP) or a nuclear run-on experiment showing decreased nascent rRNA transcription.

Response: We apologize if we did not make this clear enough in the original manuscript. This assay was previously published (Chen *et al*, 2012) and clearly shows that RNA Pol I occupancy is strongly decreased on rDNA in a H3K56A mutant. NopI has been shown to be the H2AQ105 methyltransferase. Consequently, our interest in assessing NopI occupancy at

the rDNA in wild type vs H3K56A mutants was as a means to explain decreased H2AQ105 methylation in the H3K56A mutant rather than an indirect measure of rDNA transcription. We have tried to explain this now better in the revised version of the manuscript (lines 154-156).

Line 155: hst3/4 deletion strains have higher levels of H3K56ac, but H2AQ105me levels are the same as WT. Is it surprising that H2AQ105me levels are not rising with increasing H3K56ac?

<u>Response:</u> As H3K56ac is distributed across the genome, a global increase does not necessarily mean a sharp increase of H3K56ac at the rDNA repeat. In case of hst3/4, the situation is more complex as a knock-out leads to an increase in rDNA copy number (Jack *et al*, 2015), which usually does not necessarily lead to higher levels of rDNA transcription (Ide *et al*, 2010). As pointed out in the initial version of the manuscript, H2AQ105me levels seem to correlate well with the transcriptional state of the rDNA locus, an increase in H3K56ac does not need to impact H2AQ105me levels.

4) Based on the ChIP-qPCR, H2AQ105A mutants still demonstrate 50% Nhp2 recruitment compared to WT H2AQ105. However, in vitro experiments show no binding of recombinant Nhp2 or Nhp2-myc to the Q105A mutant peptide. This raises the question of whether Nhp2 has different modes of recruitment to the rDNA, besides interacting with H2AQ105me.

<u>Response:</u> We agree with the reviewer on this point. Nhp2 has been co-purified with members of the RNA Pol I complex in a few studies, incl. RPA34 (Tarassov *et al*, 2008), RPA43 (Lebaron *et al*, 2005; Tarassov *et al*, 2008; Fath *et al*, 2000) and RPA135 (Hierlmeier *et al*, 2013) as well as the rDNA chromatin component Hmo1 (Krogan *et al*, 2006). We have pointed this out now in the discussion section of the revised manuscript (lines 205-210).

5) Based on the lane labels in Figure 4D, it appears that this assay evaluates the binding of human Nhp2 with the yeast peptide containing Q105me, rather than the human Q104me peptide. Please clarify which peptide was used in this panel. If the yeast peptide was indeed used, it is not surprising that the human Nhp2 binds weaker to the yeast Q105me peptide compared to yeast Nhp2. Unless yeast and human Nhp2 are identical at the amino acid level, caution is warranted when interpreting these results.

<u>Response:</u> The peptide sequences between yeast and human would only differ in the last two amino acids at the C-terminus of the peptide. The core binding site would be identical, Nhp2 itself is very well conserved. To help the reader interpreting the results properly, we have now included alignments of the peptide sequence and Nhp2 itself as new Figure EV 3.

6) The paper's main novelty is the identification of Nhp2 as a direct reader of H2AQ105me. However, all the data supporting this point are based on similar pull-down experiments that are coupled to mass spectrometry or PAGE/WB. Ideally, the direct interaction between Q105me and Nhp2 should be verified using a different ITC or equivalent assay.

<u>Response:</u> We agree with the reviewer and over the years we have attempted several different approaches to tackle this issue. Unfortunately, due to the hydrophobicity of the peptide, it is very difficult to bring into solution at high concentrations for any structural experiment. Using thermophoresis, we calculated the apparent dissociation constant to be

larger than 20μM, which is approaching a range that makes interpretations difficult. However, we would like to point out that this is very common for epigenetic readers that often have binding constants in the higher micromolar ranges up to ~100μM (Ruthenburg *et al*, 2011). To circumvent this problem, we approached this issue from a different angle. The NMR structure of Nhp2 showed a proline in trans configuration that was required to bind the pseudouridylation complex. Mutating the next residue – a serine (S82W) – abolished cis-/trans isomerisation of the proline and stabilized the binding to the pseudouridylation complex. To understand more of the relationship between Nhp2 binding to either H2A or H/ACA snoRNP complex, we introduced this mutant into Nhp2 and repeated the peptide pulldowns. Intriguingly, this mutation now lost the ability to distinguish between unmethylated and methylated Q105 and also bound the alanine substitution (R-Figure 2A). Subsequently, we performed ChIP-qPCR in WT and H2AQ105A strains using either WT Nhp2 or the Nhp2S82W mutation. In line with the peptide pulldown, the Nhp2S82W mutant was enriched more efficiently in the H2AQ105A strain than WT Nhp2, suggesting a loss of specificity for H2AQ105 methylation (R-Figure 2B). This suggests that the cis/trans isomerisation that plays a role in Nhp2-H/ACA binding is at least also partially important for recognition of modified and unmodified H2AQ105.

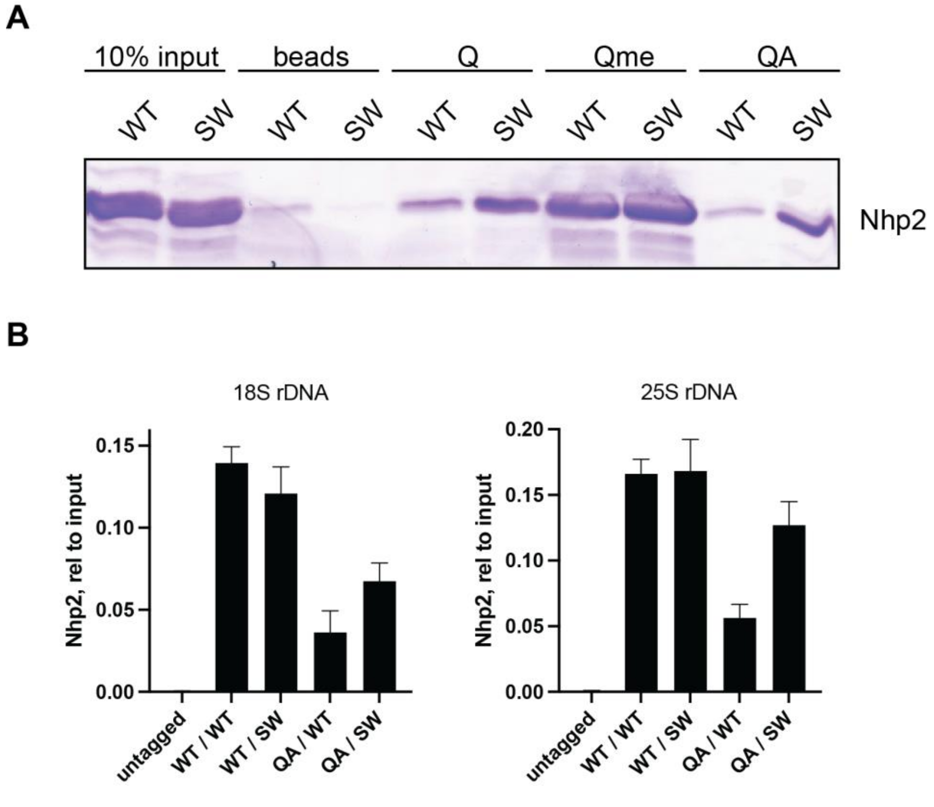

R-Figure 2: A mutant deficient in cis/trans isomerisation of proline 83 loses specificity for H2AQ105me. A) Nhp2 wildtype (WT) and Nhp2 S82W (SW), which shows stabilised binding to the H/ACA snoRNP was purified recombinantly. Subsequently, peptide pulldowns were performed using either beads alone or beads pre-bound to the indicated peptides. After extensive washing, bound Nhp2 was eluted by boiling beads in Laemmli buffer and analysed by SDS-PAGE and Coommassie staining. B) Binding of myc-tagged Nhp2 (WT) and myc-tagged Nhp2 S82W (SW) to either the 18S or 25S region of the rDNA locus was analysed by chromatin immunoprecipitation followed by qPCR. Enrichment of Nhp2 is given relative to input. N=3, error bars are SEM.

7) The authors found 17 candidate proteins (enriched in SSU) interacting with each of Nhp2 and H2AQ105me. This raises a question; Do H2AQ105A mutants have defects in the SSU processing? A Northern would show any rRNA processing defects that might arise if SSU processing enzymes (such as Mpp10, Imp4, Utp11 (line 290)) are not recruited efficiently to the pre-rRNA. Similarly, RNA IP may show if these factors' recruitment to the pre-rRNA is affected in the H2AQ105A mutants.

Response: We agree with the reviewer and attempted a RIP using Mpp10. Unfortunately, we were not able to IP any rRNA using myc-tagged Mpp10 above the untagged background. However, as outlined in our answer to question 1, we now provide new evidence that H2AQ105A mutants have a mild but significant enrichment of 18S precursors indicating that there is a defect in processing of the small rRNA subunit (new Figure 5I).

8) Recommendation. One wonders whether differential gene expression analysis with RNA-seq from WT and H2AQ105A strains would reveal compensatory transcriptomic changes and provide insight into this network.

Response: As outlined in our answer to question 1, we have now performed an RNA-seq that does show mild, compensatory transcriptional changes (new Figure 3E).

Minor comments:

-Some abbreviations are not defined throughout the text. For example, see Line 89 Qme; Figure 1B Noc, and Figure 2A NTS2, 5' ETS, ITS1. In general, the figures are poorly described in the legends. It would greatly help the audience if the authors included more details in the figure legends. For example, it's not clear what red versus grey represents in Figure 1E. What are the error bars in the ChIP-qPCR graphs (like in Figure S2)?

Response: We have now tried to define abbreviations throughout the text and provide better figure legends.

-For clarity, it would be better to abbreviate RNA Pol I with space rather than "RNA PolI," which is frequently referred to throughout the manuscript, as this might be confused with RNA pol II.

Response: We agree and have made the corresponding changes.

-Line 103-107: Please explain in the methods section how the samples were collected after being grown deep into stationary phase but then "sampled during log-phase of growth."

Response: clarified

-Line 130: "addition of rapamycin led to a loss of RNA polI from the rDNA locus" is shown in Figure S1B and not in S1A. "... and a stop in proliferation" is shown in figure S1A and not in S1B.

Response: corrected

-Figure 3C and line 195: Are the ribosome biogenesis factors enriched in certain stages of ribosome biogenesis, i.e., early vs. late? Can the authors classify them according to the complexes they belong to, such as t-UTP, UTP-B, MPP10 complex, etc?

Response: We see members of two distinc complexes here, i.e. the early (18S processing) complex Mpp10, incl. Imp4 and its assembly factor Utp11. On the other hand, we also see components of the 60S pre-ribosomal particle, such as Nog1 and Nug1 that associate later. Based on the results of the Northern, we suggest that Nhp2 at least recruits members of the early complex. We have made this clearer in the revised manuscript (lines 272-273).

-Figure 3C is not very meaningful without labels for individual proteins or networks, such as those noted in the legend.

Response: Added a label highlighting that the nodes in red belong to ribosome biogenesis factors.

-Line 230: "mammalian cell lysate." What cell type was used in these experiments? This information should be mentioned in the results as well as the methods section.

Response: Information added

-How many biological replicates were included in the mass spectrometry experiments? What about technical replicates? The methods section seems to be missing these.

Response: We have added this information to the methods section. (lines 484-487)

-What platform were the ChIP samples sequenced on (i.e., Illumina HiSeq, MiSeq, etc.)? What is the read length? Was input also sequenced? Please show the track for input in Figure 4E. The y-axis label for the ChIP-seq tracks is missing in Figure 4E. I'm wondering if the two depicted 35S rDNA copies are identical? Nhp2 coverage seems identical at the two 35S regions, whereas the H2AQ105me track looks very much different? Are the Nhp2 and H2AQ105me tracks mapped the same way, using the same parameters?

Response: We have added the missing information in the method section (HiSeq 4000). The ChIP-seq and RNA-seq data are now also uploaded to GEO with all relevant information. The H2AQ105me track in Figure 4E was taken as is from (Tessarz et al, 2014).

“ChIP-seq data was mapped to the yeast genome (R64) using bowtie2 (Langmead & Salzberg, 2012). Mapped reads were indexed and merged using samtools (Li *et al*, 2009) and converted to bigwig files using deepTools bamCoverage (v 2.0) (Ramírez *et al*, 2016) with a bin size of 10 and normalization to genomic content. Single gene tracks were generated through the IGV genome browser. Peak calling was performed using Macs2 (Zhang *et al*, 2008) with peaks displaying an FDR $< 10^{-5}$ considered statistically significant. The H2AQ105me track was directly taken from Tessarz *et al.*, 2014 (E-MTAB-1447) without any further analysis.”

We now also show an input track, which we agree is a very good control considering the problem of copy number differences in rDNA.

-Line 212: Shouldn't it refer to Figure 4B?

Response: Corrected.

Referee #2:

In this report, the authors provide evidence that the poorly understood histone modification H2AQ105me, which localizes to solely to the rDNA, is bound by the protein Nhp2 that functions in ribosomal biogenesis. They further demonstrate that H2AQ105me levels are cell-cycle dependent, and that they specifically depend on cell proliferation as arresting cells at any stage of the cell cycle reduces the global abundance of this histone modification. They further provide evidence that H2AQ105me depends on both Pol I transcription, as well as the TORC1 regulated Rtt109-Asf1 pathway, which previously was shown to regulate H3K56ac at the rDNA and promote Pol I transcription.
Overall, the work presented in this manuscript is of high quality, and the results presented would be of significant interest to the readership of EMBO Reports. However, there are a few concerns that need to be addressed before acceptance of the manuscript. These are described below.

We would like to thank the reviewer for the very positive comments on our manuscript and have answered the individual concerns below.

1) The authors discuss the requirement for cell proliferation in the maintenance of H2AQ105me, and they provide data that arresting cells in G1, early S-phase, and at the G2/M boundary reduces its abundance. While they specifically point out that a previous study demonstrated that S-phase arrest impairs Pol I transcription, they do not provide an explanation for why this modification (which seems to depend on Pol I transcriptional activity) also is reduced in G1 and/or G2/M arrested cells. Have they (or anyone else) examined the transcriptional activity of Pol I in G1 or G2/M arrested cells? This point is especially important since most anabolism occurs in G1. The authors should address and/or elaborate on this issue as it might be expected that cells in G1 would still have transcriptionally active Pol I, and hence H2AQ105me.

Response: We apologize if we have not been clearer in our manuscript. We agree that anabolism takes place in G1 and that under normal circumstances, ribosome biogenesis takes place as well. However, prolonged G1 arrest leads to a continuous opening of the rDNA locus, while it leads to lower recruitment of RNA Pol I (Wittner *et al*, 2011). In addition, there is very little rRNA synthesis in G2/M as chromatin compacts before cell division, which is visible by nascent RNA labelling experiments (Iyer-Bierhoff *et al*, 2018). We have added a discussion on this important point in the revised version of the manuscript (lines 87-90).

2) The results for Nhp2 as a binder of H2AQ105me are strong, but I do not believe they are 100% convincing. It would be really helpful if the authors actually identified the region of Nhp2 that is responsible (using their recombinant protein approach and in vitro binding as one possibility), made mutations in this region, and then expressed the mutant in vivo to test if it fails to bind to the rDNA and/or fails to pull-down H2AQ105me histone. Combined with

the data they already have presented, this would unequivocally demonstrate that Nhp2 is an in vivo binder for this epigenetic modification.

Response: We fully agree with the reviewer and would love to be able to provide this data. However, in the absence of a structure, it is very difficult to generate mutants that lose H2AQ105me binding efficiency. We attempted to generate a structure using crystallography and NMR, but both attempts were hampered by the fact that the region surrounding Q105 is hydrophobic, which prevented us from generating peptide solutions with sufficiently high concentrations for structure determination. As mentioned in our answer to reviewer 1, we tried to tackle this issue differently. The original NMR structure of Nhp2 showed a proline in trans configuration that was required to bind the pseudouridylation complex. Mutating the next residue – a serine (S82W) – abolished cis-/trans isomerisation of the proline and stabilized the binding to the pseudouridylation complex. To understand more of the relationship between Nhp2 binding to either H2A or H/ACA snoRNP complex, we introduced this mutant into Nhp2 and repeated the peptide pulldowns. Intriguingly, this mutation now lost the ability to distinguish between unmethylated and methylated Q105 and also bound the alanine substitution (R-Figure 2A, please see above). Subsequently, we performed ChIP-qPCR in WT and H2AQ105A strains using either WT or the Nhp2S82W mutation. In line with the peptide pulldown, we observed increased enrichment of this mutant at the rDNA in the H2AQ105A strain (R-Figure 2B, please see above), suggesting that the cis/trans isomerisation that plays a role in Nhp2-H/ACA binding is, at least in part, important for H2AQ105me recognition.

Technical concern:

1) Peptide pull-down experiments can be difficult to perform since normalizing the amount of each distinct peptide used for the pull-downs can be somewhat variable. This issue becomes especially important for the experiments in this report since these peptides are histone peptides, which typically are highly charged and tend to stick to proteins non-specifically. Unequal amounts of the different peptides could potentially skew the results. The authors should demonstrate for their peptide pull-down experiments equal amounts of the peptides present, at least in an "input" type format. Since the peptides are biotin-labeled, a simple dot-blot combined with streptavidin-HRP immunoblot would suffice, which would demonstrate to the reviewer and/or reader that equal amounts of the different peptides are being tested.

Response: We fully agree with the reviewer and we actually did this control as part of the experiment, but did not include the slot blots of peptides in the original version of the manuscript. In the revised version, we show the corresponding slot blots next to the peptide pulldowns.

Referee #3:

The manuscript "Nhp2 is a reader of H2AQ105me and part of a network integrating metabolism with rRNA synthesis" proposes that Nhp2 specifically recognizes H2AQ105me at rDNA as part of the rRNA synthesis pathway. The evidence that demonstrates Nhp2 and H2AQ105me interact is found in figure 4, where a H2AQ105A mutant has reduced Nhp2 binding relative to wild-type by ChIP-qPCR. Nhp2 was further shown to specifically interact

with methylated H2AQ105 proteins through peptide pulldowns. Interestingly, the authors determined that this chromatin-protein interaction was not tied to the known function of Nhp2 in pseudouridylation of rRNA, but instead propose a new role for Nhp2 in small ribosomal subunit (SSU) processing. The evidence provided in figure 5 supports the hypothesis that H2AQ105me is not essential for pseudouridylation; however, support for Nhp2's role in SSU processing is limited to analyzing mass spectrometry analyses of Nhp2 and H2AQ105me. The findings from this study would be strengthened by directly testing the hypothesis that Nhp2 reads H2AQ105me as part of the SSU processing pathway. The first 2 figures of the study focus on H2AQ105me regulation instead of the Nhp2-H2AQ105me interaction. The authors propose that H2AQ105me is cell-cycle regulated in a similar manner to H3K56 acetylation, and that H3K56 acetylation and the Tor signaling pathway are key regulators of H2AQ105me at rDNA. This data, as presented, is not strongly related to the Nhp2 finding and detracts from the overall story; however, in the discussion the authors draw an interesting parallel between H3K56 acetylation and SSU processing that, if explored, could greatly enhance their findings and focus their study. Overall, the main finding that Nhp2 reads H2AQ105me is supported by the data, but the purpose of this interaction is not adequately explored and thus it might be a bit premature to call it a bonafide reader. Additionally, the initial data characterizing H2AQ105me regulation could be more strongly tied to the main finding by exploring the relationship between H3K56 acetylation, H2AQ105me regulation, and Nhp2 function in SSU processing.

We thank the reviewer for the positive view on our manuscript and the constructive feedback. Please find a point-by-point address to the concerns below.

Major concerns:
1. The SGA analysis provided in figure 1 is poorly represented by the images shown. In the image meant to demonstrate rescue with H2AQ105A, all colonies throughout the image appear larger. Generating growth curves comparing H2AQ105A rescued candidates to WT-H2AQ105me candidates would provide more definitive evidence, however this data would not strengthen the Nhp2 hypothesis and could be removed.

<u>Response:</u> We agree with the reviewer that the effect of the H2AQ105A mutant on the slow growth phenotype is subtle. We still find it surprising that there is an obvious link to ribosome biogenesis, which ties in with the notion that H2AQ105me is connected to this pathway. However, we now performed a small-scale screen to recapitulate some candidates in an independent background (W303). We had problems generating some strains this background, but of the three we managed to generate, one (rpl12) recapitulated the results (new Figure 1G). As also reviewer 1 and the overall evaluation points to the SGA as a weakness of the manuscript without much of an addition to the overall findings, we would offer to remove these data from the manuscript.

2. As presented, the first two figures detract from the focus of the manuscript, which is the Nhp2-H2AQ105me interaction. Rather than focusing on cell-cycle regulation in figure 1, the authors could focus on the shared cell-cycling between H2AQ105me and H3K56 (which is nicely shown in figure 1 panel A) before moving into regulation of H2AQ105me by H3K56 acetylation.

Response: As pointed out in our answer to concern 1, we would be fine with removing the SGA data and combine Figures 1 and 2. We still think that showing the regulation of H2AQ105me by H3K56 acetylation is important as mutations in this pathway have been linked to processing defects of the small ribosomal subunit (Chen *et al*, 2012), which results in the accumulation of rRNA processing intermediates. As you will see in our revised manuscript, we have now added data demonstrating that H2AQ105A shows similar defects in processing of the 18S rRNA (new Figure 5I), linking the two parts of the story.

3. It is unclear how the Nop1 ChIP-qPCR data presented in figure 3 is related to the Nhp2-H2AQ105me story. This data could be removed or should be further explained.

Response: This piece of data was added to explain how H3K56A impacts H2AQ105 methylation, that is to say, how the absence of H3K56 acetylation leads to lower levels of H2AQ105me. We apologize if this was not clearly explained in the original manuscript and we have attempted to highlight this better in the revised version (lines 154-156).

4. In figure 4, the authors provide ChIP-seq data for Nhp2 and H2AQ105me. To better interpret this data, the authors should provide a scale for the Y-axis of the ChIP-seq peaks. A more detailed explanation of the experimental approach, including the number of samples sequenced and how the mapping of reads to the repetitive rDNA locus was conducted, would be beneficial. Additionally, this data prompts the question of Nhp2 binding throughout the genome. Does Nhp2 bind only to the rDNA repeats, or does it bind to other regions? Could the authors explain why there are no Nhp2 peaks at the smaller H2AQ105me peaks shown along chromosome 12?

Response: We have added a detailed explanation of the individual samples (two independent biological replicates per sample, including IgG and input) and the mapping strategy in the revised version as part of the Materials and Methods section. We have also added scales to the screen shot and added an input track to better judge enrichments. The Q105me track stems from a previous publication (Tessarz *et al*, 2014) and we have made this clear in the revised figure legend. We also now provide new supplementary information (new Appendix Table S3) that contains all peaks called for Nhp2 genome-wide together with a gene ontology enrichment for associated genes).

5. In figure 5, the authors demonstrate that Nhp2 does not interact with H2AQ105me as part of the pseudouridylation processing of rRNA. From their mass spectrometry data, they propose that Nhp2 is reading this chromatin mark to function in SSU processing. They also mention that H3K56 acetylation regulation, which they demonstrate regulates H2AQ105me in figure 2, is involved in SSU processing. If H3K56 recruitment is essential for SSU recruitment at rDNA, then the authors may have identified Nhp2's reading H3K56-regulated H2AQ105me as a key component of this pathway. The authors should examine small ribosomal subunit processing in the H2AQ105A mutant they have generated and in a Nhp2 mutant. This could be done by looking at SSU factor recruitment, such as Utp9, or by analyzing 40S levels in these two mutants.

Response: We fully agree with this point and think that this is an important experiment. We have tried various ways to perturb Nhp2 levels in the cells as a knock-out is lethal. However, we were not successful in depleting levels by either a DAMP allele or an auxin-mediated

degron system. However, we performed Northern blots using probes to measure and quantify rRNA processing defects. Indeed, we were able to detect a small but significant enrichment of 18S processing intermediates. As such a phenotype was previously described for H3K56A, this piece of data links the H3K56 acetylation pathway with H2AQ105me levels and processing of the small ribosomal subunit.

Minor concerns:
1. The qPCR data in figure 4A appears to be labeled incorrectly. The H2AQ105A bars are black but the key indicates they should be orange. This error (at least I think its an error) makes the data appear opposite of their written conclusion.

<u>Response:</u> Absolutely, we apologise and are grateful that you point out this mistake. It is corrected in the revised version.

2. In the introduction (lines 36-40), the authors imply that chromatin modifications at rDNA regulate RNA pol I. While this may be true, the example they provide (rapamycin treatment reduces both RNA pol I transcription and acetylation levels) does not support this statement. The example provided regarding H3K56 acetylation from the Chen et al 2012 study (line 156-157) better supports this statement. While the levels of RNAP1 by ChIP are clearly reduced, there is no direct measurement of transcription to support the claim.

<u>Response:</u> We have revised this part of the introduction.

3. In panel E of figure 2, the 28S line appears dashed. It is not clear if this line is dashed for a particular reason or if it should solid like the other lines.

<u>Response:</u> Corrected.

4. The authors identify five potential readers from their MS data described in figure 3. A more detailed explanation of what characteristics they used to identify potential readers would be helpful.

<u>Response:</u> We added additional explanation (line 202).

5. Line 256 should have "extent" instead of "extend."

<u>Response:</u> Corrected.

6. Line 300 should say "maintaining" instead of "maintain."

<u>Response:</u> Corrected.

7. Line 305-306 is grammatically incorrect and should be rephrased.

<u>Response:</u> Corrected.

<u>References:</u>

Chen H, Fan M, Pfeffer LM & Laribee RN (2012) The histone H3 lysine 56 acetylation pathway is regulated by target of rapamycin (TOR) signaling and functions directly in ribosomal RNA biogenesis. *Nucleic Acids Res* 40: 6534–6546

Fath S, Milkereit P, Podtelejnikov AV, Bischler N, Schultz P, Bier M, Mann M & Tschochner H (2000) Association of yeast RNA polymerase I with a nucleolar substructure active in rRNA synthesis and processing. *J Cell Biol* 149: 575–590

Hierlmeier T, Merl J, Sauert M, Perez-Fernandez J, Schultz P, Bruckmann A, Hamperl S, Ohmayer U, Rachel R, Jacob A, *et al* (2013) Rrp5p, Noc1p and Noc2p form a protein module which is part of early large ribosomal subunit precursors in S. cerevisiae. *Nucleic Acids Res* 41: 1191–1210

Ide S, Miyazaki T, Maki H & Kobayashi T (2010) Abundance of ribosomal RNA gene copies maintains genome integrity. *Science* 327: 693–696

Iyer-Bierhoff A, Krogh N, Tessarz P, Ruppert T, Nielsen H & Grummt I (2018) SIRT7-Dependent Deacetylation of Fibrillarin Controls Histone H2A Methylation and rRNA Synthesis during the Cell Cycle. *Cell Rep* 25: 2946-2954.e5

Jack CV, Cruz C, Hull RM, Keller MA, Ralser M & Houseley J (2015) Regulation of ribosomal DNA amplification by the TOR pathway. *Proc Natl Acad Sci U S A* 112: 9674–9679

Krogan NJ, Cagney G, Yu H, Zhong G, Guo X, Ignatchenko A, Li J, Pu S, Datta N, Tikuisis AP, *et al* (2006) Global landscape of protein complexes in the yeast Saccharomyces cerevisiae. *Nature* 440: 637–643

Lebaron S, Froment C, Fromont-Racine M, Rain J-C, Monsarrat B, Caizergues-Ferrer M & Henry Y (2005) The splicing ATPase prp43p is a component of multiple preribosomal particles. *Mol Cell Biol* 25: 9269–9282

Ruthenburg AJ, Li H, Milne TA, Dewell S, McGinty RK, Yuen M, Ueberheide B, Dou Y, Muir TW, Patel DJ, *et al* (2011) Recognition of a mononucleosomal histone modification pattern by BPTF via multivalent interactions. *Cell* 145: 692–706

Tarassov K, Messier V, Landry CR, Radinovic S, Serna Molina MM, Shames I, Malitskaya Y, Vogel J, Bussey H & Michnick SW (2008) An in vivo map of the yeast protein interactome. *Science* 320: 1465–1470

Tessarz P, Santos-Rosa H, Robson SC, Sylvestersen KB, Nelson CJ, Nielsen ML & Kouzarides T (2014) Glutamine methylation in histone H2A is an RNA-polymerase-I-dedicated modification. *Nature* 505: 564–568

Wittner M, Hamperl S, Stöckl U, Seufert W, Tschochner H, Milkereit P & Griesenbeck J (2011) Establishment and maintenance of alternative chromatin states at a multicopy gene locus. *Cell* 145: 543–554

1st Revision - Editorial Decision 20th Jul 2021

Dear Dr. Tessarz,

Thank you for the submission of your revised manuscript to our editorial offices. I have now received the reports from the three referees that were asked to re-evaluate your study, you will find below. As you will see, the referees now support the publication of your study. However, all referees have some remaining points and suggestions to improve the manuscript I ask you to address in a final revised version of the manuscript. Please also provide a point-by-point-response addressing the remaining concerns of the referee.

Moreover, I have these editorial requests:

- Please shorten the title to not more than 100 characters (including spaces).

- We plan to publish your manuscript in the Report format (as also indicated by you in the submission system). For a Scientific Report we require that results and discussion sections are combined in a single chapter called "Results & Discussion". Please do this for your manuscript. For more details please refer to our guide to authors:
http://www.embopress.org/page/journal/14693178/authorguide#researcharticleguide

- Please remove the Appendix legends from the main manuscript text and order the manuscript sections like this (using these section names):
Title page - Abstract - Introduction - Results + Discussion - Materials and Methods -Data availability section - Acknowledgements - Author contributions - Conflict of interest statement - References - Figure legends - Expanded View Figure legends.

- It seems there is presently no callout for Fig. 1G. Please check.

- Please make sure that the number "n" for how many independent experiments were performed, their nature (biological versus technical replicates), the bars and error bars (e.g. SEM, SD) and the test used to calculate p-values is indicated in the respective figure legends (also of the EV figures), and that statistical testing has been done where applicable. Please avoid phrases like 'independent experiment', but clearly state if these were biological or technical replicates. Please provide statistical testing for all the diagrams in Figs. 2B-E, 2H, 4A, EV1C/I, EV2, EV3 and EV5. If statistical testing was done but there is no significant difference, please also mark this in the diagrams (n.s.).

- As the Western blots shown are significantly cropped, could you provide the source data for all the blots (main, EV figures and Appendix figures - if there will be WB figures in the final Appendix file). The source data will be published in a separate source data file online along with the accepted manuscript and will be linked to the relevant figure. Please submit the source data (scans of entire blots) together with the revised manuscript. Please include size markers for scans of entire blots, label the scans with figure and panel number and send one PDF file per figure. The images for the Appendix should be combined in one file.

- Please remove the referee tokens from the data availability section of the final version of the paper and make sure that all the deposited data is public upon publication of the paper.

- Appendix tables S2, S3 and S5 are datasets and should be uploaded as such. Please upload the original excel files as datasets, using the filename Dataset EVx (also for their callouts). Please put

their legends on the first TAB of the excel file. Finally, please remove these from the Appendix and change the TOC and all the relevant callouts in the manuscript text.

In addition, I would need from you:
- a short, two-sentence summary of the manuscript (not more than 35 words).
- two to four bullet points highlighting the key findings of your study.
- a schematic summary figure (in jpeg or tiff format with the exact width of 550 pixels and a height of not more than 400 pixels) that can be used as a visual synopsis on our website.

I look forward to seeing the final revised version of your manuscript when it is ready. Please let me know if you have questions regarding the revision.

Best,

Achim Breiling
Editor
EMBO Reports

Referee #1:

The authors have addressed my original major comments. However, some points still require attention.

-Line 110: Did the authors mean "H2AQ105A" instead of "H2A"?
The new section summarizing what is known about the H2AQ105A mutant is confusing and could be written more clearly.
After adding this section, FACT is introduced twice in the manuscript now (lines 110-113 and 169-173). To eliminate redundancy, these sections should be combined in a way that doesn't interfere with the flow of the text.

-Given the difficulty of validating the results of the SGA and how its results are tangentially related to the main findings of the paper, I suggest removing these from the paper.

-Figure 3E: There is a minimal discussion on the results of the new RNA-seq data. Are expected hits among the top genes with altered expression, such as Nop1? Are genes mostly upregulated or downregulated? Naming the top hits and the extent to which they are upregulated/downregulated would make the results of the RNA-seq more interesting to the reader. Heat maps or volcano plots are useful for showing more detail.

- Even though the Northern shows subtle changes to rRNA processing, the original image should be included as part of supplemental materials. From the cartoon in Figure 5H, it is unclear which intermediate or aberrant molecule 32S may correspond to.

Referee #2:

The following sentence from the author's manuscript needs to be corrected. The authors refer to tyrosine on H3, but the manuscript they site concerns H3 threonine 11 (H3T11): "Recently, it was

shown that phosphorylation of tyrosine 11 in histone H3 was an endpoint of a signaling cascade that integrates a response to nutrient availability and ultimately, shaped chromatin to allow efficient transcription (Oh et al, 2018)."

Otherwise, I believe the authors have addressed the reviewers' criticisms adequately and the manuscript should be accepted for publication.

Referee #3:

The authors have performed experiments and re-worked the text to address reviewers' concerns. However, the new data added to directly test the model (Figure 5I) presents a subtle effect, which is somewhat unsatisfying. It would have been optimal to present more experiments testing the model with stronger outcomes to lend more confidence in the conclusions. Some journals (I don't know about EMBO) require molecular weight markers on Western blots (Figure 1A-C, 4B, 4D, 5A, 5G). If the SGA data is to remain in the manuscript, the deletions depicted in Figure 1D need to be labelled.

We do thank the referees for acknowledging our efforts to improve our manuscript. We have addressed the remaining concerns below in a point-by-point manner. Referee Comments are in italics.

Referee #1:

The authors have addressed my original major comments.

Response: We thank the reviewer for the appreciation on our efforts to address his/her points during the first round of revision.

However, some points still require attention.

-Line 110: Did the authors mean "H2AQ105A" instead of "H2A"?
The new section summarizing what is known about the H2AQ105A mutant is confusing and could be written more clearly.
After adding this section, FACT is introduced twice in the manuscript now (lines 110-113 and 169-173). To eliminate redundancy, these sections should be combined in a way that doesn't interfere with the flow of the text.

Response: As we have removed the corresponding figure from the manuscript as suggested by the reviewer (see below), this part of the manuscript was re-written to allow for a coherent and understandable flow of the manuscript.

-Given the difficulty of validating the results of the SGA and how its results are tangentially related to the main findings of the paper, I suggest removing these from the paper.

Response: As suggested by the reviewer, we have now removed the SGA results and combined remaining Figure 1 with Figure 2.

-Figure 3E: There is a minimal discussion on the results of the new RNA-seq data. Are expected hits among the top genes with altered expression, such as Nop1? Are genes mostly upregulated or downregulated? Naming the top hits and the extent to which they are upregulated/downregulated would make the results of the RNA-seq more interesting to the reader. Heat maps or volcano plots are useful for showing more detail.

Response: We have added more discussion of the RNA-seq data to the new version of the manuscript (lines 181-183 and 186-189). As mentioned in the text, there are no genes changed with a significant adjusted p-value. This is the reason we used GSEA and thus, we are not able to provide an MA- or volcano plot.

- Even though the Northern shows subtle changes to rRNA processing, the original image should be included as part of supplemental materials. From the cartoon in Figure 5H, it is unclear which intermediate or aberrant molecule 32S may correspond to.

Response: We have added one example of a scanned Northern blot as part of the new Source Data file for Figure 4. We have also tried to improve the cartoon to visualize the different intermediates in a better way.

Referee #2:

The following sentence from the author's manuscript needs to be corrected. The authors refer to tyrosine on H3, but the manuscript they site concerns H3 threonine 11 (H3T11):

"Recently, it was shown that phosphorylation of tyrosine 11 in histone H3 was an endpoint of a signaling cascade that integrates a response to nutrient availability and ultimately, shaped chromatin to allow efficient transcription (Oh et al, 2018)."

Response: We apologize for this mistake. It is now corrected in the revised version of the manuscript.

Otherwise, I believe the authors have addressed the reviewers' criticisms adequately and the manuscript should be accepted for publication.

Response: We thank the reviewer for supporting the publication of the manuscript.

\----------------
Referee #3:

The authors have performed experiments and re-worked the text to address reviewers' concerns. However, the new data added to directly test the model (Figure 5I) presents a subtle effect, which is somewhat unsatisfying. It would have been optimal to present more experiments testing the model with stronger outcomes to lend more confidence in the conclusions. Some journals (I don't know about EMBO) require molecular weight markers on Western blots (Figure 1A-C, 4B, 4D, 5A, 5G). If the SGA data is to remain in the manuscript, the deletions depicted in Figure 1D need to be labelled.

Response: We agree that it would have been optimal to report a stronger effect by H2AQ105A. However, overall, the changes (under laboratory conditions) are subtle as also seen by RNA-seq – but they collectively point to a role of H2AQ105me in rRNA processing. With respect to western blots, we have now added MW labels and provide uncropped WB as part of the Source data files. As mentioned to our answer to reviewer 1, we have followed the suggestion to remove the SGA data from the manuscript.

2nd Revision - Editorial Decision 6th Aug 2021

Peter Tessarz
Max Planck Institute for Biology of Ageing
Joseph-Stelzmann-Str. 9b
Cologne, NRW 50931
Germany

Dear Dr. Tessarz,

I am very pleased to accept your manuscript for publication in the next available issue of EMBO reports. Thank you for your contribution to our journal.

At the end of this email I include important information about how to proceed. Please ensure that you take the time to read the information and complete and return the necessary forms to allow us to publish your manuscript as quickly as possible.

As part of the EMBO publication's Transparent Editorial Process, EMBO reports publishes online a Review Process File to accompany accepted manuscripts. As you are aware, this File will be published in conjunction with your paper and will include the referee reports, your point-by-point response and all pertinent correspondence relating to the manuscript.

If you do NOT want this File to be published, please inform the editorial office within 2 days, if you have not done so already, otherwise the File will be published by default [contact: emboreports@embo.org]. If you do opt out, the Review Process File link will point to the following statement: "No Review Process File is available with this article, as the authors have chosen not to make the review process public in this case."

Should you be planning a Press Release on your article, please get in contact with emboreports@wiley.com as early as possible, in order to coordinate publication and release dates.

Thank you again for your contribution to EMBO reports and congratulations on a successful publication. Please consider us again in the future for your most exciting work.

Yours sincerely,

Achim Breiling
Editor
EMBO Reports

****

THINGS TO DO NOW:

You will receive proofs by e-mail approximately 2-3 weeks after all relevant files have been sent to our Production Office; you should return your corrections within 2 days of receiving the proofs.

Please inform us if there is likely to be any difficulty in reaching you at the above address at that time. Failure to meet our deadlines may result in a delay of publication, or publication without your corrections.

All further communications concerning your paper should quote reference number EMBOR-2021-52435V3 and be addressed to emboreports@wiley.com.

Should you be planning a Press Release on your article, please get in contact with emboreports@wiley.com as early as possible, in order to coordinate publication and release dates.

EMBO PRESS

YOU MUST COMPLETE ALL CELLS WITH A PINK BACKGROUND

PLEASE NOTE THAT THIS CHECKLIST WILL BE PUBLISHED ALONGSIDE YOUR PAPER

Corresponding Author Name: Peter Tessarz
Journal Submitted to: EMBO Reports
Manuscript Number: EMBOR-2021-52435

Reporting Checklist For Life Sciences Articles (Rev. June 2017)

This checklist is used to ensure good reporting standards and to improve the reproducibility of published results. These guidelines are consistent with the Principles and Guidelines for Reporting Preclinical Research issued by the NIH in 2014. Please follow the journal's authorship guidelines in preparing your manuscript.

A- Figures
1. Data
The data shown in figures should satisfy the following conditions:

- the data were obtained and processed according to the field's best practice and are presented to reflect the results of the experiments in an accurate and unbiased manner.
- figure panels include only data points, measurements or observations that can be compared to each other in a scientifically meaningful way.
- graphs include clearly labeled error bars for independent experiments and sample sizes. Unless justified, error bars should not be shown for technical replicates.
- if n< 5, the individual data points from each experiment should be plotted and any statistical test employed should be justified
- Source Data should be included to report the data underlying graphs. Please follow the guidelines set out in the author ship guidelines on Data Presentation.

2. Captions
Each figure caption should contain the following information, for each panel where they are relevant:

- a specification of the experimental system investigated (eg cell line, species name).
- the assay(s) and method(s) used to carry out the reported observations and measurements
- an explicit mention of the biological and chemical entity(ies) that are being measured.
- an explicit mention of the biological and chemical entity(ies) that are altered/varied/perturbed in a controlled manner.

- the exact sample size (n) for each experimental group/condition, given as a number, not a range;
- a description of the sample collection allowing the reader to understand whether the samples represent technical or biological replicates (including how many animals, litters, cultures, etc.).
- a statement of how many times the experiment shown was independently replicated in the laboratory.
- definitions of statistical methods and measures:
  - common tests, such as t-test (please specify whether paired vs. unpaired), simple χ2 tests, Wilcoxon and Mann-Whitney tests, can be unambiguously identified by name only, but more complex techniques should be described in the methods section;
  - are tests one-sided or two-sided?
  - are there adjustments for multiple comparisons?
  - exact statistical test results, e.g., P values = x but not P values < x;
  - definition of 'center values' as median or average;
  - definition of error bars as s.d. or s.e.m.

Any descriptions too long for the figure legend should be included in the methods section and/or with the source data.

In the pink boxes below, please ensure that the answers to the following questions are reported in the manuscript itself. Every question should be answered. If the question is not relevant to your research, please write NA (non applicable). We encourage you to include a specific subsection in the methods section for statistics, reagents, animal models and human subjects.

USEFUL LINKS FOR COMPLETING THIS FORM

http://www.antibodypedia.com
http://1degreebio.org
http://www.equator-network.org/reporting-guidelines/improving-bioscience-research-repor
http://grants.nih.gov/grants/olaw/olaw.htm
http://www.mrc.ac.uk/Ourresearch/Ethicsresearchguidance/Useofanimals/index.htm
http://ClinicalTrials.gov
http://www.consort-statement.org
http://www.consort-statement.org/checklists/view/32-consort/66-title
http://www.equator-network.org/reporting-guidelines/reporting-recommendations-for-tumo
http://datadryad.org
http://figshare.com
http://www.ncbi.nlm.nih.gov/gap
http://www.ebi.ac.uk/ega
http://biomodels.net/
http://biomodels.net/miriam/
http://jjj.biochem.sun.ac.za
http://oba.od.nih.gov/biosecurity/biosecurity_documents.html
http://www.selectagents.gov/

B- Statistics and general methods

Please fill out these boxes (Do not worry if you cannot see all your text once you press return)

1.a. How was the sample size chosen to ensure adequate power to detect a pre-specified effect size?	all experiments were performed using 3 independent biological replicates, except for ribosome slippage experiment, in which 8 independent experiments were performed. No power calculation was performed.
1.b. For animal studies, include a statement about sample size estimate even if no statistical methods were used.	no animal experiments involved
2. Describe inclusion/exclusion criteria if samples or animals were excluded from the analysis. Were the criteria pre-established?	no samples were excluded from analysis
3. Were any steps taken to minimize the effects of subjective bias when allocating animals/samples to treatment (e.g. randomization procedure)? If yes, please describe.	N/A
For animal studies, include a statement about randomization even if no randomization was used.	N/A
4.a. Were any steps taken to minimize the effects of subjective bias during group allocation or/and when assessing results (e.g. blinding of the investigator)? If yes please describe.	no blinding was performed
4.b. For animal studies, include a statement about blinding even if no blinding was done	N/A
5. For every figure, are statistical tests justified as appropriate?	yes
Do the data meet the assumptions of the tests (e.g., normal distribution)? Describe any methods used to assess it.	as we report biochemical/molecular data, usually three biological experiments were performed, which is insufficient to measure normal distribution. As it is standard in the field to assume near-to normal distribution, we used ttest where appropriate.
Is there an estimate of variation within each group of data?	no

Is the variance similar between the groups that are being statistically compared?	yes

C- Reagents

6. To show that antibodies were profiled for use in the system under study (assay and species), provide a citation, catalog number and/or clone number, supplementary information or reference to an antibody validation profile. e.g., Antibodypedia (see link list at top right), 1DegreeBio (see link list at top right).	H2AQ105me: Tessarz et al., Nature 2014; H3K56ac (Active Motif - 39281), G6PDH (Sigma - A9521) and c-Myc (9e10, Sigma - MABE282), FLAG (Sigma - F3165), H2AQ105me from (Tessarz et al, 2014), H2A (Abcam - ab13923), H3 (Abcam - ab1791), Utp11L (GeneTex GTX115929), Dyskerin (Santa Cruz sc-373956) and human Nhp2 (Proteintech 15128-1-AP).
7. Identify the source of cell lines and report if they were recently authenticated (e.g., by STR profiling) and tested for mycoplasma contamination.	HEK293T were bought from ATCC and tested for mycoplasma

* for all hyperlinks, please see the table at the top right of the document

D- Animal Models

8. Report species, strain, gender, age of animals and genetic modification status where applicable. Please detail housing and husbandry conditions and the source of animals.	N/a
9. For experiments involving live vertebrates, include a statement of compliance with ethical regulations and identify the committee(s) approving the experiments.	N/A
10. We recommend consulting the ARRIVE guidelines (see link list at top right) (PLoS Biol. 8(6), e1000412, 2010) to ensure that other relevant aspects of animal studies are adequately reported. See author guidelines, under 'Reporting Guidelines'. See also: NIH (see link list at top right) and MRC (see link list at top right) recommendations. Please confirm compliance.	N/A

E- Human Subjects

11. Identify the committee(s) approving the study protocol.	N/A
12. Include a statement confirming that informed consent was obtained from all subjects and that the experiments conformed to the principles set out in the WMA Declaration of Helsinki and the Department of Health and Human Services Belmont Report.	N/A
13. For publication of patient photos, include a statement confirming that consent to publish was obtained.	N/A
14. Report any restrictions on the availability (and/or on the use) of human data or samples.	N/A
15. Report the clinical trial registration number (at ClinicalTrials.gov or equivalent), where applicable.	N/A
16. For phase II and III randomized controlled trials, please refer to the CONSORT flow diagram (see link list at top right) and submit the CONSORT checklist (see link list at top right) with your submission. See author guidelines, under 'Reporting Guidelines'. Please confirm you have submitted this list.	N/A
17. For tumor marker prognostic studies, we recommend that you follow the REMARK reporting guidelines (see link list at top right). See author guidelines, under 'Reporting Guidelines'. Please confirm you have followed these guidelines.	N/A

F- Data Accessibility

18: Provide a "Data Availability" section at the end of the Materials & Methods, listing the accession codes for data generated in this study and deposited in a public database (e.g. RNA-Seq data: Gene Expression Omnibus GSE39462, Proteomics data: PRIDE PXD000208 etc.) Please refer to our author guidelines for 'Data Deposition'.<br><br>Data deposition in a public repository is mandatory for:<br>a. Protein, DNA and RNA sequences<br>b. Macromolecular structures<br>c. Crystallographic data for small molecules<br>d. Functional genomics data<br>e. Proteomics and molecular interactions	The mass spectrometry proteomics data have been deposited to the ProteomeXchange Consortium via the PRIDE partner repository with the dataset identifier PXD023510. RNA- and ChIP-seq data is available at gene expression omnibus, accession number: GSE176302.
19. Deposition is strongly recommended for any datasets that are central and integral to the study; please consider the journal's data policy. If no structured public repository exists for a given data type, we encourage the provision of datasets in the manuscript as a Supplementary Document (see author guidelines under 'Expanded View' or in unstructured repositories such as Dryad (see link list at top right) or Figshare (see link list at top right).	see 18
20. Access to human clinical and genomic datasets should be provided with as few restrictions as possible while respecting ethical obligations to the patients and relevant medical and legal issues. If practically possible and compatible with the individual consent agreement used in the study, such data should be deposited in one of the major public access-controlled repositories such as dbGAP (see link list at top right) or EGA (see link list at top right).	N/A
21. Computational models that are central and integral to a study should be shared without restrictions and provided in a machine-readable form. The relevant accession numbers or links should be provided. When possible, standardized format (SBML, CellML) should be used instead of scripts (e.g. MATLAB). Authors are strongly encouraged to follow the MIRIAM guidelines (see link list at top right) and deposit their model in a public database such as Biomodels (see link list at top right) or JWS Online (see link list at top right). If computer source code is provided with the paper, it should be deposited in a public repository or included in supplementary information.	N/A

G- Dual use research of concern

22. Could your study fall under dual use research restrictions? Please check biosecurity documents (see link list at top right) and list of select agents and toxins (APHIS/CDC) (see link list at top right). According to our biosecurity guidelines, provide a statement only if it could.	N/A
